# DNA Methyltransferase Inhibition Upregulates the Costimulatory Molecule ICAM-1 and the Immunogenic Phenotype of Melanoma Cells

**DOI:** 10.1016/j.xjidi.2024.100319

**Published:** 2024-10-16

**Authors:** Alessandra S.P. Cereghetti, Patrick Turko, Phil Cheng, Stephan Benke, Ala’a Al Hrout, Andreas Dzung, Reinhard Dummer, Michael O. Hottiger, Richard Chahwan, Lorenza P. Ferretti, Mitchell P. Levesque

**Affiliations:** 1 Department of Dermatology, University Hospital of Zurich, University of Zurich, Schlieren, Switzerland; 2 Flow Cytometry Facility, University of Zurich, Zurich, Switzerland; 3 Institute of Experimental Immunology, University of Zurich, Zurich, Switzerland; 4 Department of Molecular Mechanisms of Disease, University of Zurich, Zurich, Switzerland

**Keywords:** DNA methyltransferase, DNMTi, Epigenetics, ICAM-1, Melanoma

## Abstract

In cutaneous melanoma, epigenetic dysregulation is implicated in drug resistance and tumor immune escape. However, the epigenetic mechanisms that influence immune escape remain poorly understood. To elucidate how epigenetic dysregulation alters the expression of surface proteins that may be involved in drug targeting and immune escape, we performed a 3-dimensional surfaceome screen in primary melanoma cultures and identified the DNA-methyltransferase inhibitor decitabine as significantly upregulating the costimulatory molecule ICAM-1. By analyzing The Cancer Genome Atlas melanoma dataset, we further propose ICAM-1 upregulation on melanoma cells as a biomarker of a proinflammatory and antitumorigenic signature. Specifically, we showed that DNA-methyltransferase inhibitor administration upregulated the expression of the antigen-presenting machinery, HLA class I/II, as well as the secretion of the proinflammatory chemokines CXCL9 and CXCL10. Our in silico analysis on The Cancer Genome Atlas and ex vivo experiments on human primary melanoma samples revealed that increased ICAM-1 expression positively correlated with increased immunogenicity of human melanoma cells and correlated with increased immune cell infiltration. These findings suggest a therapeutic approach to modulate the immunogenic phenotype of melanoma cells, hence supporting the exploration of DNA-methyltransferase inhibitor as a potential inducer of infiltration in immunologically cold tumors.

## Introduction

Point mutations of the serine/threonine kinases *BRAF* (V600) and *NRAS* (Q61) of the MAPK pathway are the most frequent drivers in cutaneous melanoma (CM), the deadliest type of cutaneous tumor (Gutiérrez-Castañeda et al, 2020; Paluncic et al, 2016; Sullivan and Flaherty, 2013). Currently, one of the first-line therapies for the treatment of *MAPK*-mutated unresectable CM is the administration of inhibitors targeting the aberrant protein BRAFV600E (BRAF inhibitor) and/or its downstream target MAPK/extracellular signal–regulated kinase kinase (MEK) (MEK inhibitor), but there are no approved therapies directed against altered epigenetic states in patients with melanoma (Aloia et al, 2019; Flavahan et al, 2017). However, intrinsic or acquired resistance limits 5-year overall survival after treatment with MAPK inhibitor (MAPKi) to 34% (Robert et al, 2019). This underlies the need for alternative treatment strategies for patients with MAPKi-resistant CM that may possibly target aberrant epigenetic states. Indeed, growing evidence supports the involvement of a dysregulated melanoma epigenome in tumor suppressor silencing, in the maintenance of drug-resistant and senescent states, in downregulating immunogenicity, and in specifying the preferred metastatic site (Berglund et al, 2020; Liu et al, 2019; Madorsky Rowdo et al, 2020; Micevic et al, 2017; Orozco et al, 2018; Strub et al, 2020). Among epigenetic modifications, DNA methylation is a hallmark of malignant melanoma (Emran et al, 2019; Lian et al, 2012; Saghafinia et al, 2018). Indeed, DNA methylation drives melanoma immunoediting, in which low immunogenic clones are selected upon prolonged exposure to immune effector cells, hence leading to significant dampening of antitumor immunity (Germenis and Karanikas, 2007; Tucci et al, 2019). Despite aberrant DNA methylation being a prognostic marker for the progression of various cancer types besides melanoma, administration of DNA methyltransferase inhibitors (DNMTis) is approved exclusively for myelodysplastic syndromes and pancreatic cancer (Baer et al, 2022; Wachtel et al, 2008; Wouters et al, 2017). To date, DNMTi-mediated modulation of the epigenome of advanced CM has not extensively been described, nor have we reached a deep understanding of the epigenetic-induced regulation of melanoma treatment resistance or its immune escape. To elucidate the clinical potential of targeting the epigenome of resistant CM and to identify epigenetically modified surface features of resistant melanoma cells, we performed an epigenetic drug screening of cell surface markers of 3-dimensional (3D) melanoma cultures. Indeed, we focused initially on the major surface markers—regrouped under the term surfaceome—owing to their importance in mediating the interactions with the immune effector cells and dictating the immunogenicity of the melanoma cells. In this study, we show that the DNMTis upregulated the ICAM-1, a ligand mediating the interaction with several immune effector cells. Particularly, ICAM-1 upregulation on melanoma cells positively correlated with increased density of CD8^+^ T cell and other immune cells and with an IFN-γ gene signature predictive for improved response to therapy. Moreover, DNA-methylation inhibition increased CXCL9/CXCL10 secretion, overexpression of major histocompatibility complex class I/II antigens and components of the antigen-processing machinery, thus suggesting the augmentation of melanoma immunogenicity.

## Results

### DNMTi modulates melanoma surfaceome by upregulating the costimulatory molecule ICAM-1

**Figure 1:**
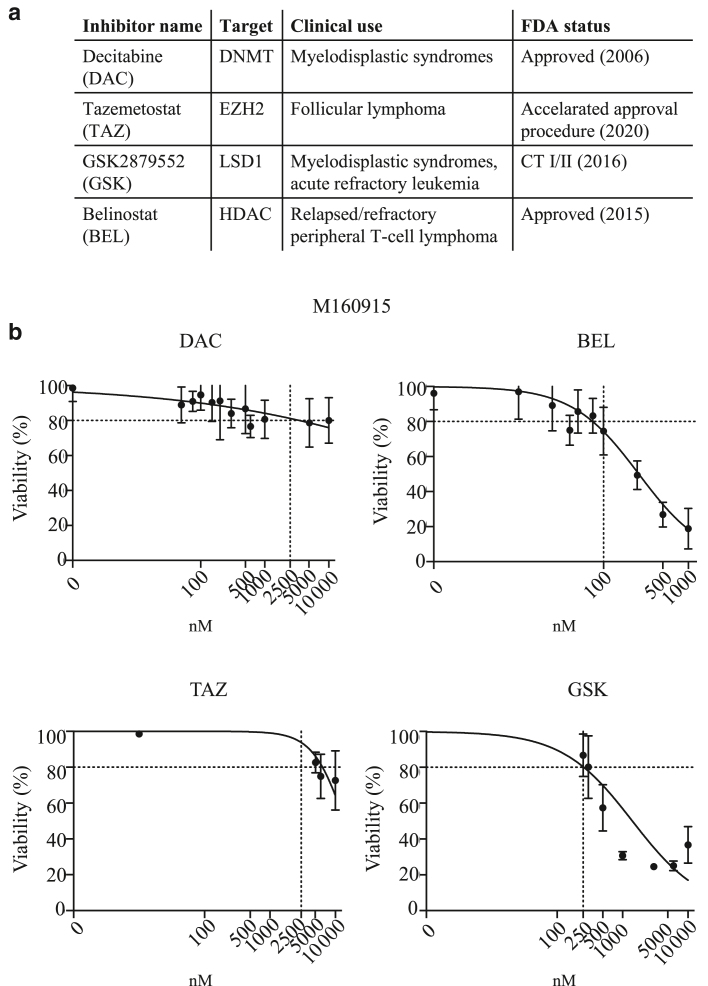
**Epigenetic inhibitor selection for the surfaceome screen.** (**a**) Selected epigenetic inhibitors for the surfaceome screen. **(b)** Dose–response curve of M160915 after administration of DAC (0–10,000 nM), BEL (0–1000 nM), TAZ (0–10,000 nM), or GSK (0–250 nM) for 72 hours. Residual viability was quantified by a Resazurin assay, according to the manufacturer’s instructions. The final epigenetic inhibitor doses corresponding to 80% viability inhibition (dashed line) were selected for the surfaceome screen. BEL, belinostat; DAC, decitabine; FDS, Food and Drug Administration; GSK, GSK2879552; TAZ, tazemetostat.

**Figure 2:**
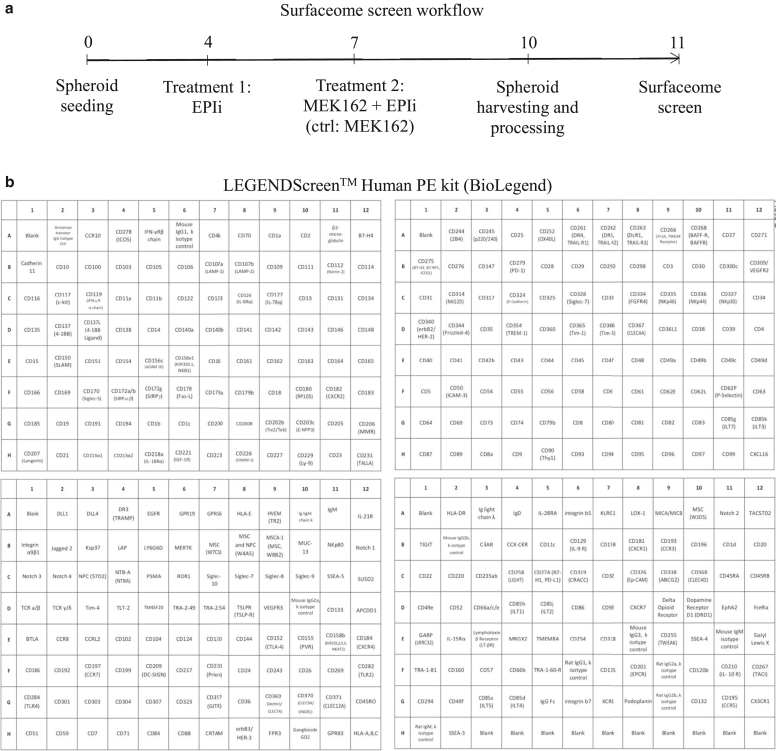
**Surfaceome screen workflow and targets.(a)** Patient-derived M160915 melanoma spheroids were treated with the epigenetic inhibitors (denoted as EPIi) DAC (2.5 μM), TAZ (3 μM), BEL (100 nM), or GSK (250 nM) on day 4. EPIi treatment was repeated on day 7 and combined with MEK162 (100 nM). Control spheroids were treated on day 4 with a corresponding dilution of drug solvent (DMSO) and on day 7 with MEK162 as noted earlier. On day 10, melanoma spheroids were dissociated, and single cells were counted and stained with a fixable life–dead dye. Treatment-specific barcoding was generated by staining CD44 coupled with a treatment-specific fluorophore. Cells were pooled, aliquoted into the four 96-well plates of the LEGENDScreen Human PE kit (BioLegend), stained, and fixed, according to the manufacturer’s instructions. Fluorescence intensity of 361 surface proteins was evaluated by fluorescence-based quantification using a BD LSRFortessa cytometer (BD Biosciences). **(b)** Overview of the 361 surface proteins analyzed in the surfaceome screen. BEL, belinostat; DAC, decitabine; GSK, GSK2879552; MEK, MAPK/extracellular signal–regulated kinase kinase; TAZ, tazemetostat.

**Figure 3:**
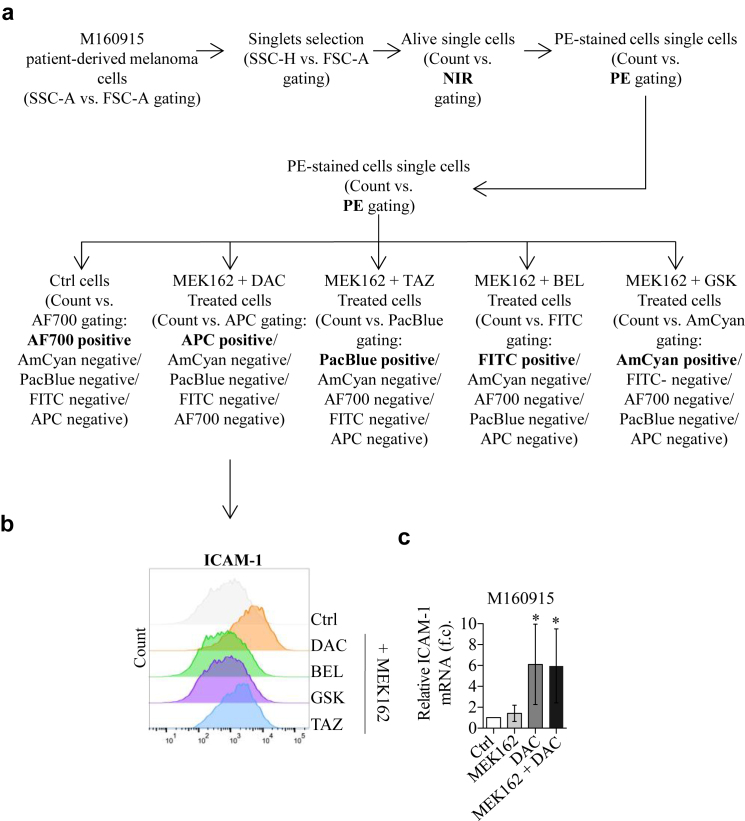
**Gating and debarcoding strategies used for surfaceome screen.** (**a**) Gating strategy used for debris exclusion (FSC-A vs SSC-A), doublet exclusion (FSC-A vs FSC-H), dead cell exclusion (NIR^+^) and selection of protein X–expressing cells (PE^+^) in the surfaceome screen. **(b)** Treatment-specific CD44-based debarcoding was performed by taking advantage of the mutual gate exclusion FlowJo function (indicated by an ∗) where CD44-AF700^+^ = MEK162-treated cells, CD44-AmCyan^+^ = MEK162 + GSK–treated cells, CD44-PacBlue^+^ = MEK162 + TAZ–treated cells, CD44-FITC^+^ = MEK162-BEL–treated cells, and CD44-APC^+^ = MEK162-DAC–treated cells. For each treatment, PE^+^ cells were plotted individually for analysis of treatment effect on each specific marker. **(c)** Representative histogram plot of PE^+^ signal intensity (log_10_ fluorescence scale) representing treatment-dependant ICAM-1 expression. APC, allophycocyanin; BEL, belinostat; Ctrl, control; DAC, decitabine; FSC-A, forward scatter area; FSC-H, forward scatter height; GSK, GSK2879552; MEK, MAPK/extracellular signal–regulated kinase kinase; PE, phycoerythrin; SSC-A, side scatter area; TAZ, tazemetostat.

**Figure 4:**
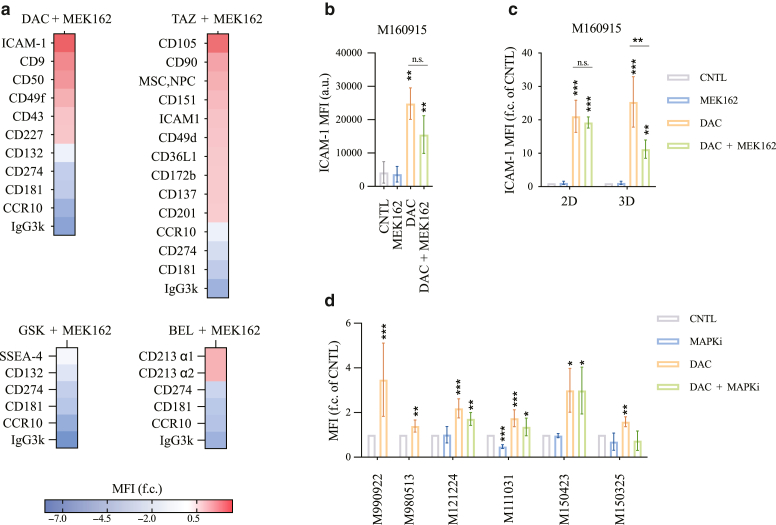
**DNMTi modulates melanoma surfaceome by upregulating the costimulatory molecule ICAM-1.** (**a**) Differentially expressed surface proteins in M160915 spheroids after treatment with MEK162 (100 nM) and DAC (2.5 μM) or TAZ (3 μM) or GSK (250 nM) or BEL (100 nM) for 144 hours. Protein levels were measured by flow cytometry. Statistically significant hits after evaluation with Welch’s *t*-test are depicted as MFI log_2_ fold change of MEK162-treated (denoted as MFI f.c.). **(b)** ICAM-1 protein levels after treatment of M160915 spheroids with DAC ± MEK162 or drug vector for 144 hours as in **a**. ICAM-1 levels were measured by flow cytometry, and data are depicted as MFI a.u. and depicted as mean ± SD (n ≥ 6). Statistically significant differences were evaluated by 1-way ANOVA with posthoc Dunnett’s multiple comparison correction. **(c)** ICAM-1 protein levels measured by flow cytometry in M160915 2-dimensional or spheroids after treatment with DAC ± MEK162 as in **a**. Statistically significant differences were evaluated by 2-way ANOVA with posthoc Sidak’s multiple comparison correction. Data are depicted as MFI log_2_ fold change of Ctrl mean ± SD (n = 3). **(d)** ICAM-1 protein levels were measured by flow cytometry in several 2-dimensional cultures of human MAPKi-resistant or -sensitive melanoma cell lines after treatment with DAC (2.5 μM) (+MAPKi for MAPKi-resistant cell lines: 100 nM MEK162 for M150325, 100 nM LGX818 and M121224 and both for M111031, M150423) for 144 hours. Data are depicted as MFI log_2_ fold change of Ctrl mean ± SD (n ≥ 2). Statistically significant differences were evaluated as in **a** except for M990922 and M980513, which were subjected to paired *t*-test for all panels: ∗*P* ≤ .05, ∗∗*P* ≤ .01, and ∗∗∗*P* ≤ .001. a.u., arbitrary unit; BEL, belinostat; Ctrl, control; DAC, decitabine; DNMTi, DNA methyltransferase inhibitor; GSK, GSK2879552; MAPKi, MAPK inhibitor; MEK, MAPK/extracellular signal–regulated kinase kinase; MFI, median fluorescence intensity; TAZ, tazemetostat.

**Figure 5:**
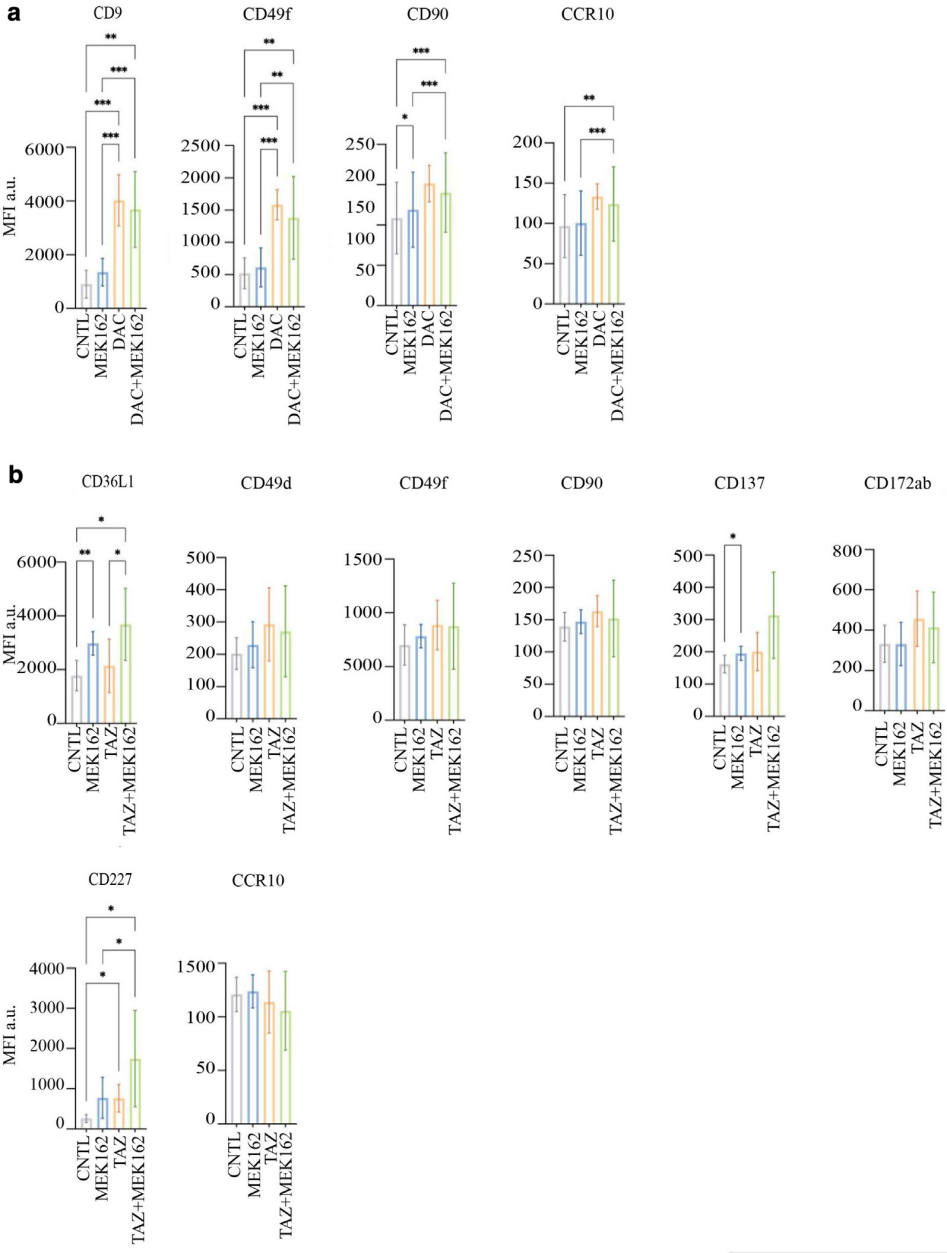
**Validation of surfaceome screen hits.** (**a, b**) Protein expression validation of significant hits in M160915 spheroids after treatment with (**a**) DAC (2.5 uM) ± MEK162 (100 nM) or (**b**) TAZ (3 μM) ± MEK162 (100 nM) for 144 hours. Protein levels were measured by flow cytometry, and data are depicted as mean ± SD (n ≥ 3). Statistically significant differences were evaluated by 1-way ANOVA with posthoc Dunnett’s multiple comparison correction: ∗*P* ≤ .05, ∗∗*P* ≤ .01, and ∗∗∗*P* ≤ .001. DAC, decitabine; MEK, MAPK/extracellular signal–regulated kinase kinase; TAZ, tazemetostat.

**Figure 6:**
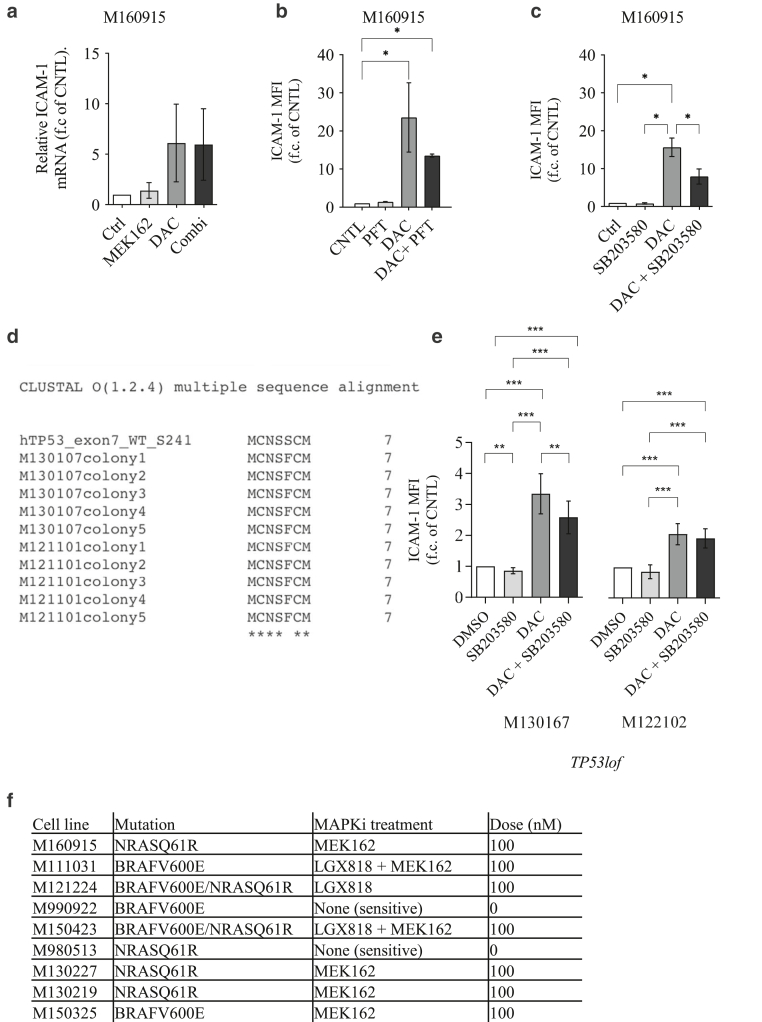
**DAC induction of ICAM-1 partially relies on p53 and p38/MAPK activation.** (**a**) Quantification of *ICAM1* mRNA induction in M160915 cells after treatment with DAC (2.5 uM) alone or in combination with MEK162 (100 nM) or single controls for 72 hours by qRT-PCR. Relative gene expression quantification is depicted as function of mean ± SD delta cycle threshold (2exp^-(ΔΔCt)^). (**b, c**) Patient-derived M160915 was treated with DAC (5 μM) alone or combined with the (**b**) p53 inhibitor pifithrin-α (denoted as PFT, 2.5 μM) or the (**c**) p38-inhibitor SB203580 (1 uM) for 72 hours. ICAM-1 protein expression was quantified by fluorescence-based analysis and is depicted as MFI fold change (denoted as MFI f.c.) of control cells. **(d)** Detection of the homozygous *TP53* loss-of-function point-mutation S241F in M130107 and M121101. Multiple sequence alignment of single-allele protein sequences was generated using the free online tool Clustal Omega (https://www.ebi.ac.uk/Tools/msa/clustalo/). Fully conserved amino acids are indicated with ∗. For each cell line, 5 protein alignments are depicted. **(e)** ICAM-1 quantification in patient-derived *TP53*^−/−^ cells after treatment with DAC (2.5 μM) and/or SB203580 (1 uM) as in **a**. **(f)** Cell-line−specific MAPKi doses used for all experiments. For all panels, data are depicted as mean ± SD (n ≥ 3). Statistically significant differences were evaluated by 1-way ANOVA with posthoc Dunnett’s multiple comparison correction: ∗*P* ≤ .05, ∗∗*P* ≤ .01, and ∗∗∗*P* ≤ .001. DAC, decitabine; MAPKi, MAPK inhibitor; MEK, MAPK/extracellular signal–regulated kinase kinase; MFI, median fluorescence intensity.

Owing to the importance of epigenetic (dis)regulation in modulating the interactions between melanoma cells and the immune effector cells, we designed a drug screening in 3D melanoma cultures to identify cell surface markers altered by epigenetic inhibitors. To this end, we performed a surfaceome screen on MEK inhibitor–resistant *NRAS*-mutated patient-derived CM cells (M160915) cultured as 3D spheroids. Epigenetic inhibitors were selected according to the following criteria: (i) residual viability after epigenetic treatment ≥ 80%; (ii) approval/ongoing evaluation by the Food and Drug Administration for any other pathology or accelerated approval process ongoing; and (iii) inhibitor target is one of the main classes of epigenetic modifications: DNA methylation, histone methylation, or histone acetylation (Figure 1a and b). M160915 cells were grown as spheroids and treated with binimetinib (MEK162) alone or in combination with decitabine (DAC), tazemetostat, GSK2879552 (denoted as GSK), or belinostat as indicated in Figure 2a. Cell surface epitope expression was analyzed by flow cytometry using a cell surface marker screening kit containing phycoerythrin-conjugated antibodies against 361 cell surface epitopes (Figure 2b). To discriminate each condition, M160915 single-cell suspension obtained after spheroid disaggregation were labeled with a CD44-targeting antibody coupled to a treatment-specific fluorophore and then stained for the antibody panel (Figure 3a and b). Median fluorescence intensities (MFIs) for each phycoerythrin-conjugated antibody were measured and normalized to MEK162-treated control cells (Figure 3c). Interestingly, with a *P* ≤ .05 and log_2_ MFI fold change > 1 or < (–1), the expression of 10 cell surfaces protein was significantly altered after DAC + MEK162 treatment, 13 after tazemetostat + MEK162 treatment, and 5 after belinostat + MEK162 or GSK + MEK162 treatment (Figure 4a). Owing to the 1-shot nature of this experiment, no statistical analysis could be performed on the results of the primary surfaceome screening; Therefore, to exclude any possible false-positive result and identify the strongest induction, these preliminary data were then re-evaluated in subsequent validation rounds. This allowed us to identify the 10 proteins (3% of the analyzed surfaceome cluster) for which expression was significantly upregulated in response to either DAC or tazemetostat with MEK162 coadministration (Figures 4b and Figure 5). Among the validated hits, DAC treatment showed the most consistent and strongest upregulation of the costimulatory glycoprotein ICAM-1 (Figure 4b). Interestingly, the DAC-induced ICAM-1 expression observed in M160915 3D-cultured spheroid was further validated in M160915 adherent 2-dimensional culture (Figure 4c). Because of this observation and owing to the high degree of complexity of 3D culturing, all the subsequent experiments were performed in 2 dimensional. DNMTi also induced *ICAM-1* mRNA increase (Figure 6a). We then validated our results of DAC-induced ICAM-1 upregulation in 6 additional patient-derived melanoma cell cultures (Figure 4d), regardless of the MAPK-sequence variant status or the resistance profile (please refer to Figure 6f for the treatments used). This allowed us to determine whether DAC-induced ICAM-1 upregulation was associated with MAPKi resistance. Interestingly, the detected increase in ICAM-1 expression was not further enhanced by cotreatment with MEK162, LGX818, or both, indicating that in this system, this phenomenon is solely driven by DNA-methylase inhibition. In addition, we could also demonstrate that ICAM-1 expression partially relied on DAC-induced p53 and p38 activation (Figure 6b–e), which were previously described to be distinct regulators of ICAM-1 expression (Gorgoulis et al, 2003; Hsu et al, 2011). Collectively, these results show that among the selected subset of melanoma surfaceome proteins, DNMTi-induced ICAM-1 upregulation is the most strongly modulated epitope after administration of several epigenetic inhibitors targeting the main epigenetic mark classes.

### DNMTi impairs proliferation and induces apoptosis in MAPKi-resistant cell lines

**Figure 7:**
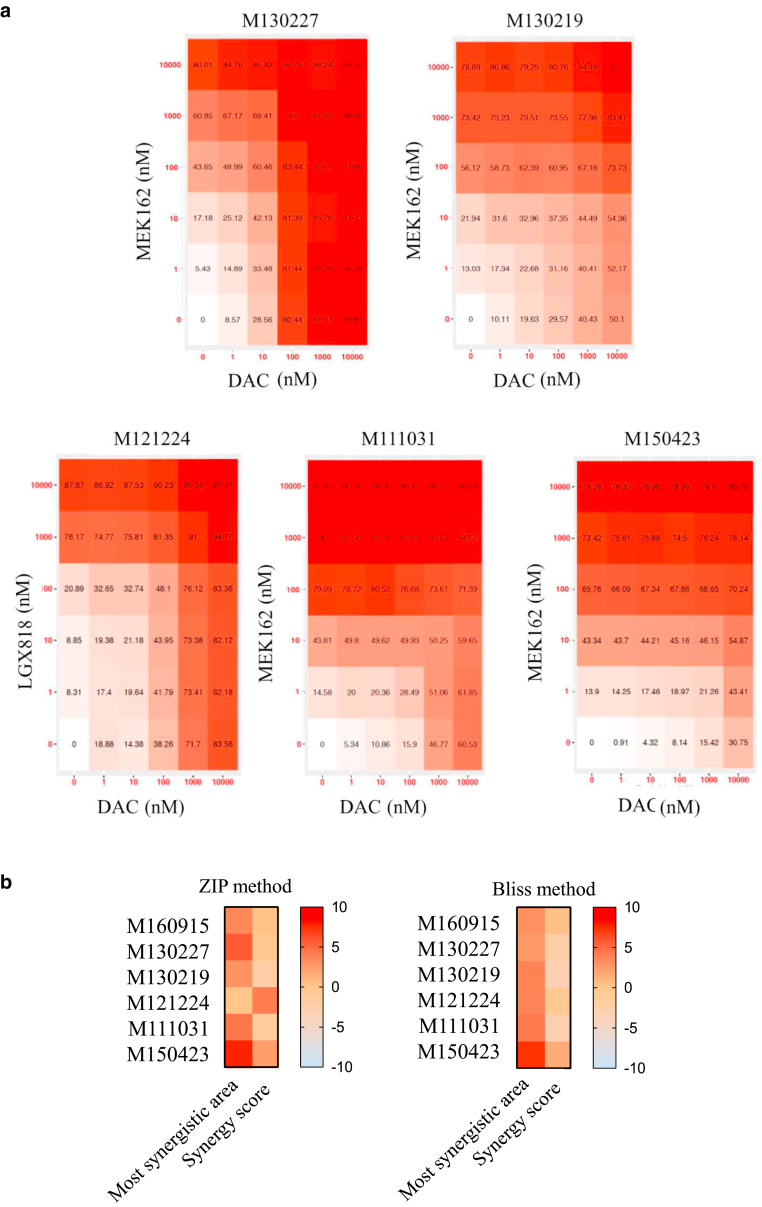
**Viability of MAPKi-resistant cell lines is additively reduced by DAC + MAPKi treatment.** (**a**) Drug interaction matrices of selected MAPKi-resistant cell lines depicting the viability inhibition (%) after coadministration of MAPKi and DNMTi (0–10,000 nM) on day 4 and day 7 after seeding (Figure 6 provides the details on the cell-line–specific MAPKi treatment). Residual cell viability was measured on day 10 after seeding by Resazurin assay. Data are depicted as mean viability inhibition fractions (%) (n = 4). Drug interaction matrices were generated using the free online software SynergyFinder2.0 (https://synergyfinder.fimm.fi). **(b)** Drug interaction score heat maps depicting the most synergistic area and the mean synergy score for each cell line tested in **a**. Data were fitted using the Loess approach. Synergy was calculated using the ZIP (left) or the Bliss (right) models after algorithm-based outlier correction. Synergy scores (–10 < x < 10) indicate an addictive drug interaction. Data are depicted as raw mean (n = 4). DAC, decitabine; DNMTi, DNA methyltransferase inhibitor; MAPKi, MAPK inhibitor.

**Figure 8:**
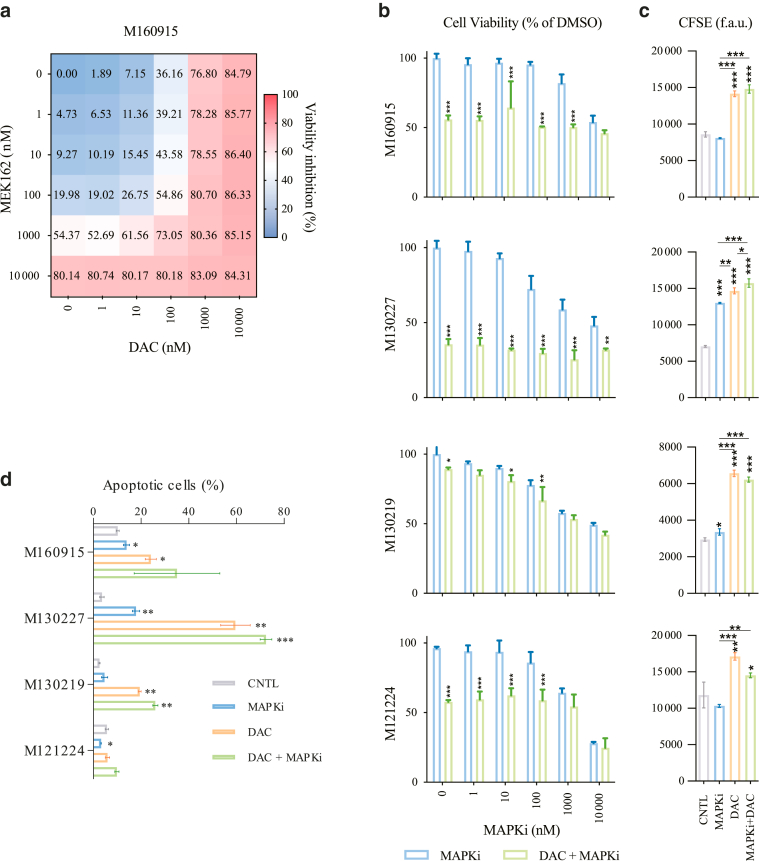
**DNMTi impairs proliferation and induces apoptosis in MAPKi-resistant cell lines.** (**a**) Viability inhibition matrix of M160915 after coadministration of DAC ± MEK162 (0–10,000 nM). Residual cell viability was measured on day 10 after seeding by Resazurin assay (n = 4). **(b)** Viability quantification after treatment of several cell lines with MAPKi (100 nM MEK162 for M160915, M130227, and M130219 or 100 nM LGX818 for M121224) ± DAC (2.5 μM) for 72 hours by Resazurin assay. Data are depicted as mean ± SD (n = 3). Statistically significant differences were evaluated by 2-way ANOVA with posthoc Sidak multiple comparison correction (compared with the CNTL [control]). **(c)** CFSE intensity signal after treatment as in **b** was measured by fluorescence-based quantification. MFI of carboxyfluorescein succinimidyl ester (CFSE) data are depicted as mean ± SD (n = 3) (MFI a.u.). Statistically significant differences were evaluated by 1-way ANOVA with posthoc Dunnett’s multiple comparison correction. **(d)** Apoptotic cell fraction quantification after treatment as in **c**. Statistically significant differences were evaluated by 1-way ANOVA with posthoc Dunnett’s multiple comparison correction (compared with the CNTL). For all panels, ∗*P* ≤ .05, ∗∗*P* ≤ .01, and ∗∗∗*P* ≤ .001. a.u., arbitrary unit; CFSE, carboxyfluorescein succinimidyl ester; DAC, decitabine; DNMTi, DNA methyltransferase inhibitor; MAPKi, MAPK inhibitor; MEK, MAPK/extracellular signal–regulated kinase kinase; MFI, median fluorescence intensity.

**Figure 9:**
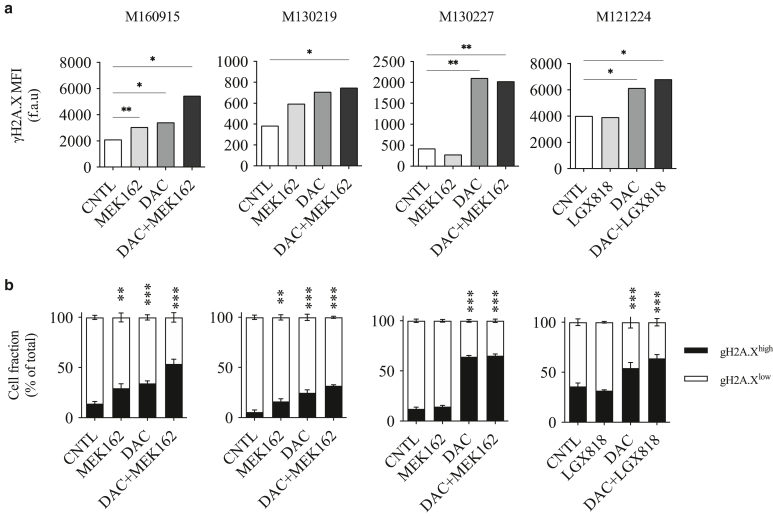
**Coadministration of DAC and MAPKi increases DNA damage in MAPKi-resistant patient-derived melanoma cell lines.** (**a, b**) Induction of γ H2A.X DNA-damage foci after treatment of MAPKi-resistant cell lines as indicated with DAC (2.5 uM) and MEK162 (100 nM) or LGX818 (100 nM) for 72 hours was evaluated by fluorescence-based quantification. γ H2A.X MFI was evaluated **(a)** on the single-cell level or **(b)** as γ H2A.X^high^ versus γ H2A.X^low^ fractions of the total cell population. Data are depicted as MFI fold change of control (MFI f.c.) or percentage of total cell numbers as mean ± SD (n = 3). Statistically significant differences were evaluated by 1-way ANOVA with posthoc Dunnett’s multiple comparison correction (for **a**) or by 2-way ANOVA with posthoc Sidak multiple comparison correction (compared with the CNTL). For all panels, ∗*P* ≤ .05, ∗∗*P* ≤ .01, and ∗∗∗*P* ≤ .001. DAC, decitabine; MAPKi, MAPK inhibitor; MEK, MAPK/extracellular signal–regulated kinase kinase; MFI, median fluorescence intensity.

Altering DNA methylation induces genotoxic stress and subsequently cytotoxicity due to DNA-damage repair machinery failure and thus chromosomal instability (Bull et al, 2021). To evaluate any interaction between DNMTi and MAPKi leading to cells toxicity, combinatorial and synergistic effects of DAC and MAPKi were scored in several melanoma cells resistant to MAPKi (Figure 7a). Drugs interactions can be assessed with different mathematical methods, such as the ZIP or the Bliss independence (Yadav et al, 2015). For all cell lines tested, including the screening cell line (M160915), DAC and MAPKi coadministration (MEK162 and/or LGX818) revealed an additive effect, as indicated by the calculated ZIP and Bliss synergy score (Figures 7b and 8a), thus revealing that the combination of DAC and MAPKi may have clinical therapeutic benefit in resistant cells. Remarkably, decreased viability of M160915 cells upon DAC treatment correlated with reduced proliferation, as indicated by increased carboxyfluorescein succinimidyl ester (CFSE) retention (Figure 8b and c), and increased apoptosis (Figure 8d). Moreover, DAC treatment also induced phosphorylation H2A.X, a commonly accepted DNA damage marker, hence indicating that DNMTi induced genotoxic stress and subsequently melanoma cell death (Figure 9a and b). Collectively, these results indicate that DNMTi induced a significant apoptotic response in MAPKi-resistant cells, thus revealing the susceptibility of MAPKi-resistant melanoma cells to hypomethylating agents.

### DNMTi improves secretion of T helper 1 cell chemokines by melanoma cells

**Figure 10:**
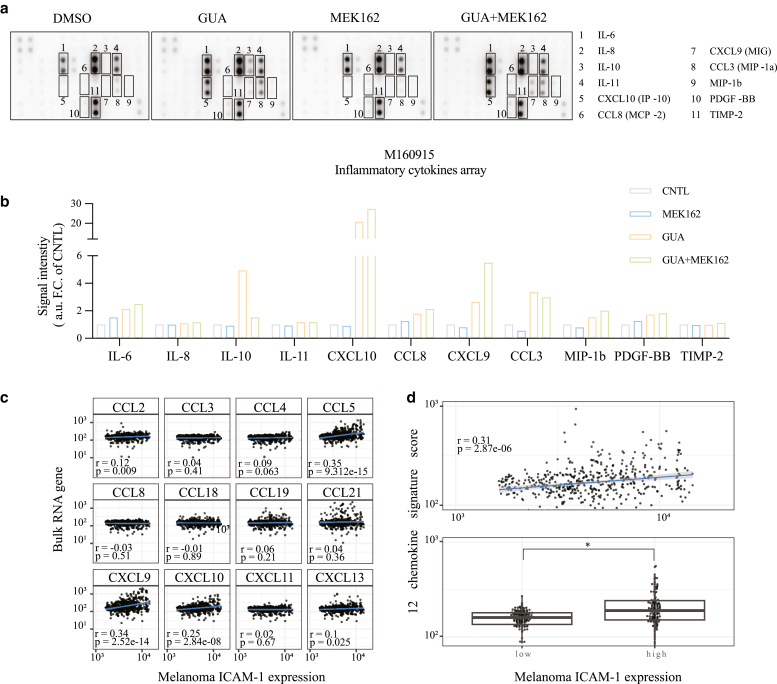
**DNMTi improves the secretion of Th1 cell chemokines by melanoma cells.** (**a**) Proinflammatory cytokine array membranes probed with supernatant isolated from M160915 cultures after treatment with GUA (2.5 μM) ± MEK162 (100 nM) for 72 hours. **(b)** Signal intensity quantification of array membranes shown in **a**. Raw signal intensities were measured using the free software ImageJ and normalized against internal controls and Ctrl signal. **(c, d)** Pearson’s correlation coefficient (r) analysis (**c**) between the melanoma ICAM-1 expression and the 12-chemokine gene signature for each gene or (**d**) between the global signature in the melanoma ICAM-1^high^ versus ICAM-1^low^ cohorts in the bulk scRNAseq TCGA SKCM data (top panel) or in the melanoma ICAM-1^high^ versus ICAM-1^low^ cohorts (bottom panel). For the comparison between ICAM-1^high^ and ICAM-1^low^ cohorts, a 1-way ANOVA test with posthoc Dunnett’s multiple comparison correction was performed. For all panels, ∗*P* ≤ .05. Ctrl, control; DNMTi, DNA methyltransferase inhibitor; GUA, guadecitabine; MEK, MAPK/extracellular signal–regulated kinase kinase; scRNAseq, single-cell RNA sequencing; SKCM, skin cutaneous melanoma; TCGA, The Cancer Genome Atlas; Th1, T helper 1.

**Figure 11:**
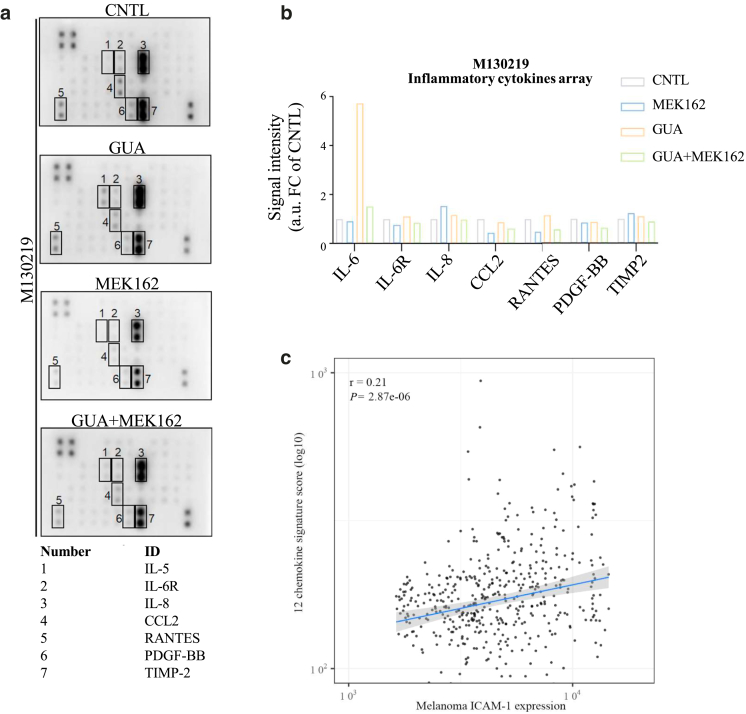
**Inhibition of DNA methylation improves the secretion of Th1 cell chemokines in M160915 (but not in M130219).** (**a**) Proinflammatory cytokine membrane arrays probed with supernatant isolated from M130219 cultures after treatment with GUA (2.5 μM) and/or MEK162+LGX818 (100 nM) for 72 hours. **(b)** Signal intensity quantification of cytokine secretion after M130219 treatment as in **a**. Data are depicted as normalized signal intensity fold change from control (left) or as raw signal (right) after pixel intensity quantification using the free software ImageJ. **(c)** Pearson’s correlation coefficient (r) analysis between continuous melanoma ICAM-1 expression and the global 12-chemokine gene signature in the bulk scRNAseq TCGA SKCM data (∗*P* ≤ .05). GUA, guadecitabine; MEK, MAPK/extracellular signal–regulated kinase kinase; scRNAseq, single-cell RNA sequencing; SKCM, skin cutaneous melanoma; TCGA, The Cancer Genome Atlas; Th1, T helper 1.

In addition to its surfaceome, melanoma secretome also modulates the tumor microenvironment (TME) composition and regulates the infiltration of immune cells (Hong et al, 2011; Zhu et al, 2021). Importantly, a recent report described a strong correlation between cytokine secretion and global genomic demethylation in patients with melanoma, suggesting a possible regulatory role of DNA methylation on the melanoma secretome (Jung et al, 2019). Therefore, we hypothesized that besides an epigenetic modulation of the surfaceome, an epigenetic-driven modulation of the melanoma secretome may also be a crucial factor in the shaping of the melanoma TME. To test this hypothesis, we quantified the secretion of the main proinflammatory cytokines after treatment of MAPKi resistant cells with the DNMTi guadecitabine (GUA), a more stable DAC prodrug (Daher-Reyes et al, 2019). In M160915, GUA induced strong upregulation of the T-cell chemokines CXCL9 and CXCL10 (Figure 10a and b), which were previously reported to be expressed in highly T-cell–infiltrated melanoma (Harlin et al, 2009). Interestingly, induction of both chemokines was even stronger upon GUA + MEK162 coadministration, suggesting a regulatory synergistic effect. Remarkably, we observed a positive correlation between increased expression of ICAM-1 and *CXCL9* (*r* = 0.34, *P* = 2.52e^14^) and *CXCL10* (*r* = 0.25, *P* = 2.84e^–8^) expressions in the public RNA-sequencing dataset from the patients with melanoma (skin cutaneous melanoma [SKCM]) in The Cancer Genome Atlas (TCGA) as well as with other components of the 12-chemokine melanoma signature (Figure 10c). Interestingly, this 12-chemokine melanoma signature was previously shown to be predictive of an increased immune infiltration and improved survival of patients with melanoma (Li et al, 2022; Messina et al, 2012). Next, we stratified the TCGA SCKM patient cohort according to ICAM-1 expression level on melanoma cells and compared the highest (ICAM-1^high^) with the lowest (ICAM-1^low^) quartiles. This revealed a significantly stronger correlation with the 12-chemokine signature in the ICAM-1^high^ subset (Figure 10d). In a second patient-derived cell line, administration of DNMTi affected the secretion of other chemokines, indicating that the effect on CXCL9/CXCL10 secretion is dependent on DNA methylation patterns of the patient (Figure 11). Collectively, these results describe that DNMTi-induced ICAM-1 upregulation correlates with a modulation of melanoma secretome in inducing the secretion of the T-cell chemokines CXCL9 and CXCL10 and with the survival positive predictor 12-chemokine melanoma signature.

### Melanoma ICAM-1^high^ expression positively correlates with increased density of M1 macrophages, T cells, and an IFN-γ gene signature in the TCGA SKCM

**Figure 12:**
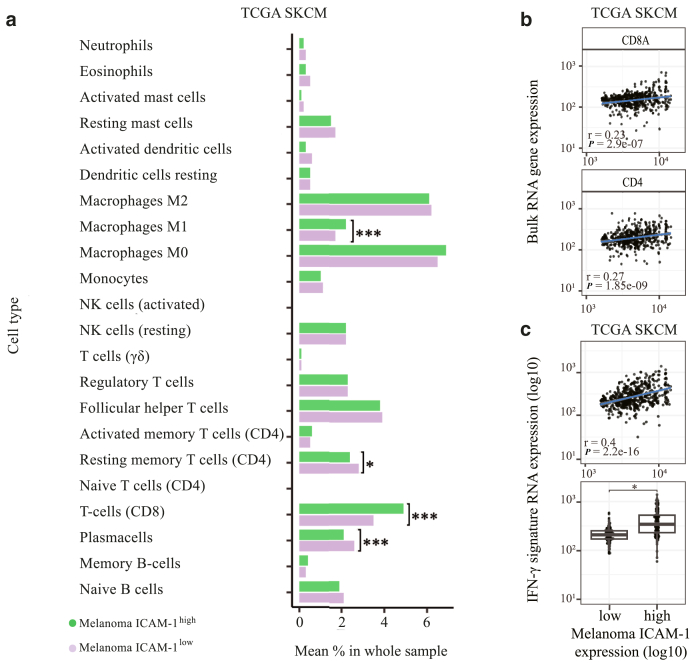
**Melanoma ICAM-1^high^ expression positively correlates with increased density of M1 macrophages, T cells, and IFN-γ gene signature in the TCGA SKCM.** (**a**) Frequency quantification of immune cell subtypes using the LMM2 immune cell reference in ICAM-1^high^ versus ICAM-1^low^ cohorts of the TCGA SKCM cohort. Data were deconvoluted to infer ICAM-1 melanoma cell expression. Correlation coefficients and correlation *P*-value are depicted. **(b, c)** Pearson’s correlation analysis between melanoma ICAM-1 expression and (**b**) CD4^+^/CD8^+^ T-cell infiltration or (**c**) *IFN*γ gene expression signature in the bulk scRNA data of the TCGA SKCM (top panel) or in the melanoma ICAM-1^high^ versus ICAM-1^low^ cohorts (bottom panel). Statistically significant differences were evaluated by paired *t*-test. For all panels, ∗*P* ≤ .05, ∗∗*P* ≤ .01, and ∗∗∗*P* ≤ .001. SKCM, skin cutaneous melanoma; TCGA, The Cancer Genome Atlas.

**Figure 13:**
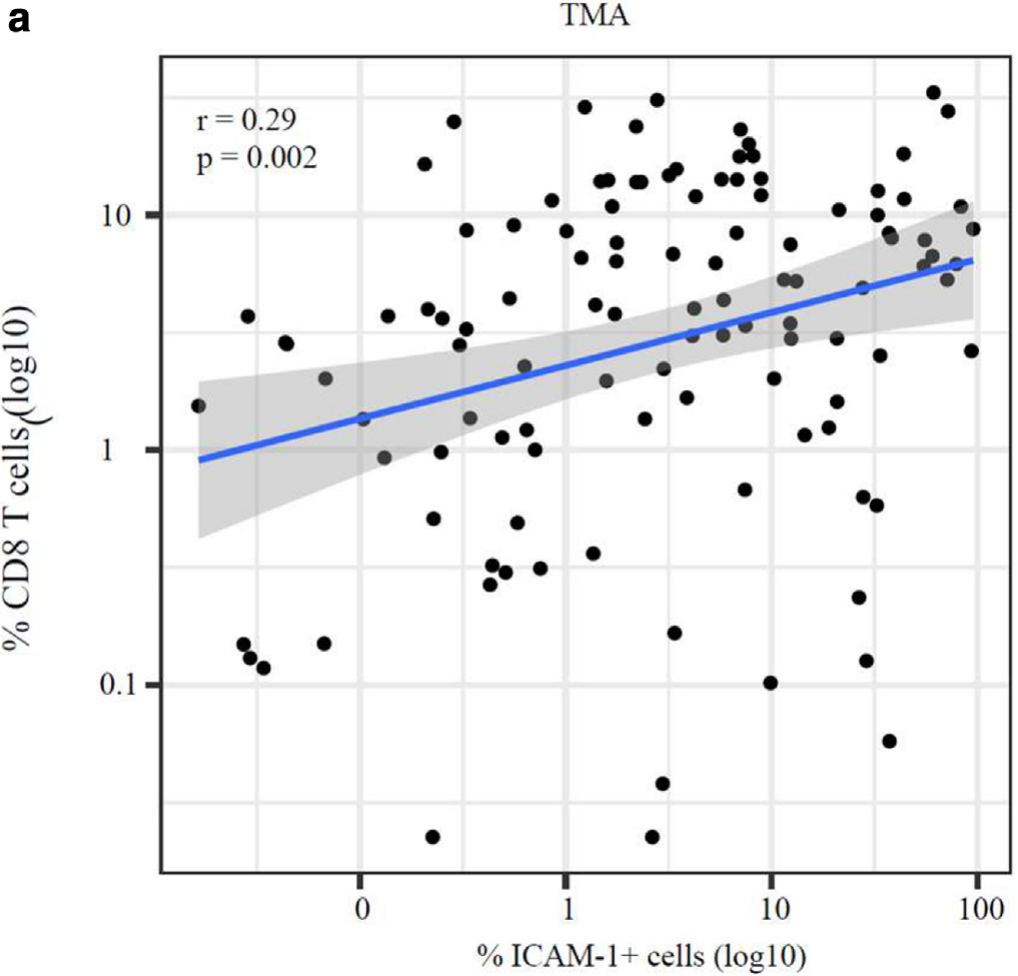
**Melanoma ICAM-1 expression correlates with decreased melanoma cell frequency and increased macrophage infiltration in the TCGA SKCM.** (**a**) Pearson’s correlation analysis of the co-occurrence of ICAM-1^+^ cells and CD8^+^ cells in a tissue microarray composed of advanced melanoma samples collected at the baseline, on therapy, or after discontinuation of ICBs (University Hospital Zurich, Zurich, Switzerland) (∗*P* ≤ .05). ICB, immune checkpoint blocker; SKCM, skin cutaneous melanoma; TCGA, The Cancer Genome Atlas; TMA, tissue microarray.

**Figure 14:**
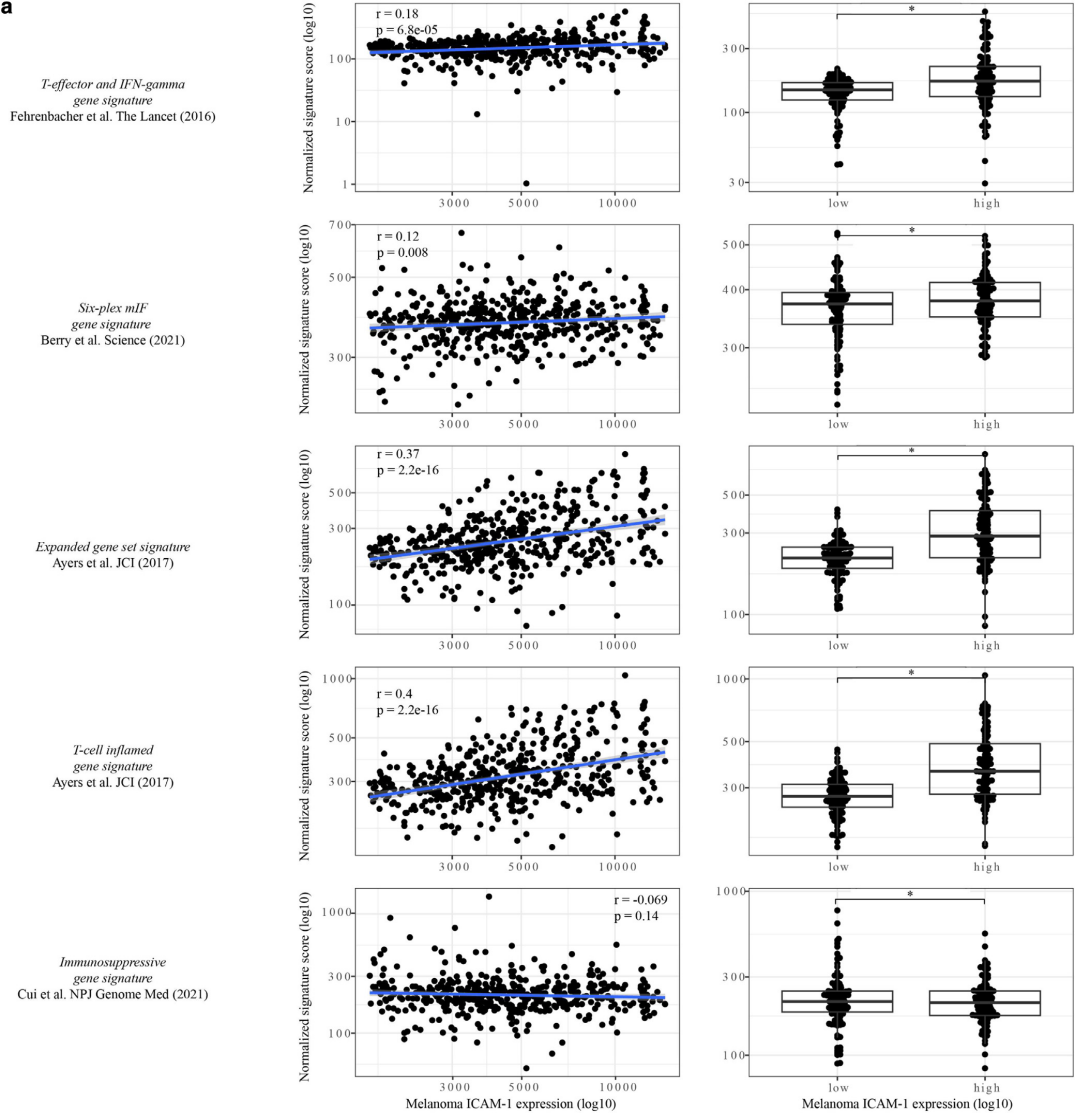
**Melanoma ICAM-1 upregulation correlates with several gene signatures predictive for T-cell infiltration and response to therapy.** (**a**) Pearson’s correlation analysis between selected gene signatures and ICAM-1 expression on melanoma cells in the bulk RNA-sequencing data (left column) or in ICAM-1^high^ versus ICAM-1^low^ cohorts (right column) of the TCGA SKCM. Sample preprocessing of bulk RNA-sequencing data was normalized by converting raw reads to counts per million. Housekeeper genes used for expression normalization were *ABCF1*, *DNAJC14*, *ERCC3*, *G6PD*, *GUSB*, *MRPL19*, *NRDE2*, *OAZ1*, *POLR2A*, *PSMC4*, *PUM1*, *SDHA*, *SF3A1*, *STK11IP*, *TBC1D10B*, *TBP*, *TFRC*, *TLK2*, *TMUB2*, and *UBB*. The geometric mean expression of housekeeper genes was calculated for each sample and was subtracted from the expression of all other genes. Single-gene expression and global signature scores for each gene signature were inferred per each cell type (Cibersortx). For each signature, the combined gene signature score was calculated by taking the geometric mean of the expression of all genes in the sample and correlated with ICAM-1 expression. Pearson’s correlation coefficient (r) and correlation *P*-value are depicted. For the comparison between the ICAM-1^high^ and ICAM-1^low^ cohorts, a 1-way ANOVA test with posthoc Dunnett’s was performed. Multiple comparison correction was performed, and a *P* ≤ .05 was considered statistically significant (∗). SKCM, skin cutaneous melanoma; TCGA, The Cancer Genome Atlas.

Because we demonstrated that DNMTi-induced ICAM-1 expression correlated with proinflammatory cytokine secretion, we further investigated whether it could be predictive of expression profiles of genes involved in immune response. To do so, we investigated this correlation in the ICAM-1^high^ versus ICAM-1^low^ TCGA SKCM cohorts generated earlier using the LM22 immune cell signature reference, an immune-specific gene list reference for identification of immune cells in bulk RNA-sequencing datasets (Prummer et al, 2022^1^). This revealed a significant positive correlation between melanoma ICAM-1^high^ expression and increased infiltration of antitumorigenic M1 macrophages and CD8^+^ T cells and a negative correlation with infiltration of plasma cells and resting CD4^+^ T-regulatory cells (Figure 12a and b). Furthermore, we analyzed co-occurrence of CD8^+^ T cells and ICAM-1^+^ cells in a tissue microarray generated from patients’ biopsies prior and after immune checkpoint blockade (University Hospital Zurich, Zurich, Switzerland), confirming a positive correlation between ICAM-1 expression and CD8^+^ T-cell infiltration (Figure 13). Because our results showed that ICAM-1 upregulation may be predictive not only of a proinflammatory melanoma secretome but also of an immunogenic TME, we then investigated whether ICAM-1^high^ levels correlated with improved prognosis of patients with melanoma. To this aim, we analyzed the relationship between ICAM-1 expression in the TCGA melanoma samples and several immune response gene signatures (Supplementary Table S1). The strongest association observed was between melanoma ICAM-1^high^ expression and the IFN-γ gene signature (Figures 12c and 14a). Upregulation of the IFN-γ gene signature was previously shown to be predictive for an improved systemic treatment response in patients with melanoma (Ayers et al, 2017). Taken together, these data show that DNMTi-induced DNA demethylation may be associated with an upregulation of antitumorigenic immune subpopulations and that melanoma ICAM-1^high^ expression may be a predictive factor for an immune-permissive TME, characterized by increased infiltration of M1 macrophages, CD8^+^ and CD4^+^ T cells, and high expression of the IFN-γ gene signature.

### DNMTis upregulate the expression of several components of the antigen-presenting machinery in MAPKi-resistant melanoma cells

**Figure 15:**
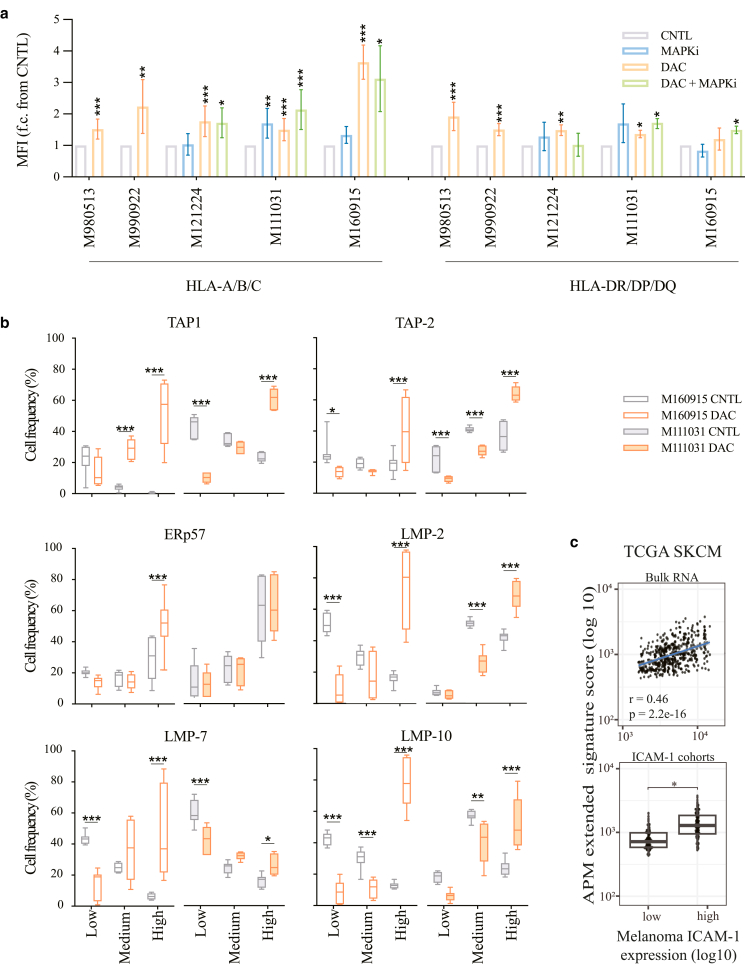
**DNMTi upregulates the expression of several components of the APM in MAPKi-resistant melanoma cells.** (**a**) Quantification of selected HLA I and HLA II protein expression after treatment with MAPKi (100 nM MEK162 for M160915, 100 nM LGX818 for M121224, and both for M111031) ± DAC (2.5 μM) or M990922 and M980513 with DAC only for 72 hours (please refer to Figure 6f for the cell line-specific treatments). Data are depicted as MFI fold change of Ctrl mean ± SD (n ≥ 3). Statistically significant differences were evaluated by 1-way ANOVA with posthoc Dunnett’s multiple comparison correction (compared with the CNTL) except for M990922 and M980513, which were subjected to paired *t*-test. *P* < .001. **(b)** Quantification of TAP1/2, LMP2/7/10, and ERp57 expression by M160915 and M111031 after treatment as in **a** using the free software QuPath-0.2.3 (https://qupath.github.io). Protein expression evaluation was based on arbitrary cut offs (low: no staining to faint staining; medium: faint to medium staining; high: medium to high staining). The low/medium/high expression cut offs were arbitrarily chosen for each marker according to the staining intensity and were kept constant across the analysis of the biological and technical replicates. For each sample, constant square regions (3–4 according to the sample size) were randomly selected for the protein expression evaluation. Data are depicted as mean ± SD (n ≥ 8). Statistically significant differences were evaluated 2-way ANOVA with posthoc Sidak’s multiple comparison correction. *P* < .001. **(c)** Pearson’s correlation coefficient (r) analysis between the global extended APM and HLA gene signature and ICAM-1 expression on melanoma cells in the bulk RNA-sequencing data (top panel) or in ICAM-1^high^ versus ICAM-1^low^ cohorts (bottom panel) of the TCGA SKCM. Data were deconvoluted to infer ICAM-1 melanoma cells. Correlation coefficients and correlation *P*-value are depicted. For all panels, ∗*P* ≤ .05, ∗∗*P* ≤ .01, and ∗∗∗*P* ≤ .001. APM, antigen-presenting machinery; Ctrl, control; DAC, decitabine; DNMTi, DNA methyltransferase inhibitor; MAPKi, MAPK inhibitor; MEK, MAPK/extracellular signal–regulated kinase kinase; MFI, median fluorescence intensity; SKCM, skin cutaneous melanoma; TCGA, The Cancer Genome Atlas.

**Figure 16:**
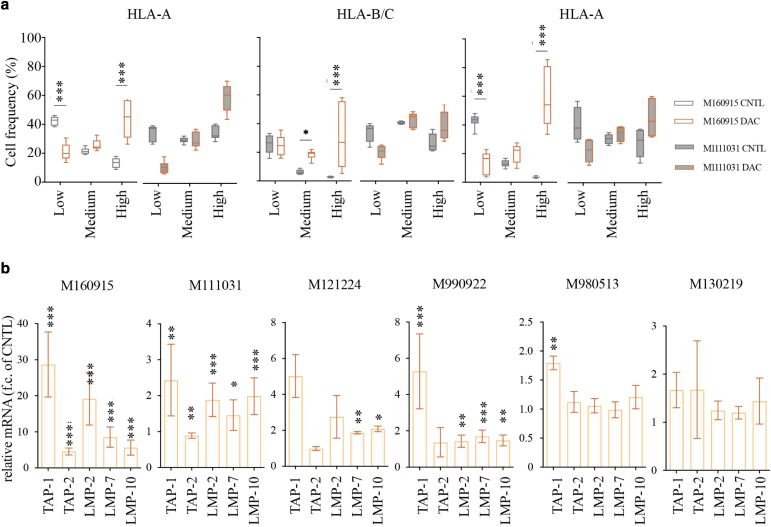
**DNMTi upregulates RNA expression of several APM components in patient-derived melanoma cells.** (**a**) Quantification of HLA-A, HLA-B, and B2M expression by M160915 (left column) or M111031 (right column) after DAC (2.5 μM) treatment for 72 hours. Protein quantification was assessed using the free software QuPath-0.2.3 (https://qupath.github.io). Data are depicted as mean ± SD (n ≥ 8). Statistically significant differences were evaluated by 2-way ANOVA with posthoc Sidak’s multiple comparison correction. **(b)** Relative mRNA quantification of selected APM after treatment as in **a** by RT-quantitative PCR. Data are depicted as function of mean ± SD delta cycle threshold (2exp^–(ΔΔCt)^) (n ≥ 3). Statistically significant differences were evaluated by 1-way ANOVA with posthoc Dunnett’s multiple comparison correction: ∗*P* ≤ .05, ∗∗*P* ≤ .01, and ∗∗∗*P* ≤ .001. APM, antigen processing machinery; DAC, decitabine; DNMTi, DNA methyltransferase inhibitor.

**Figure 17:**
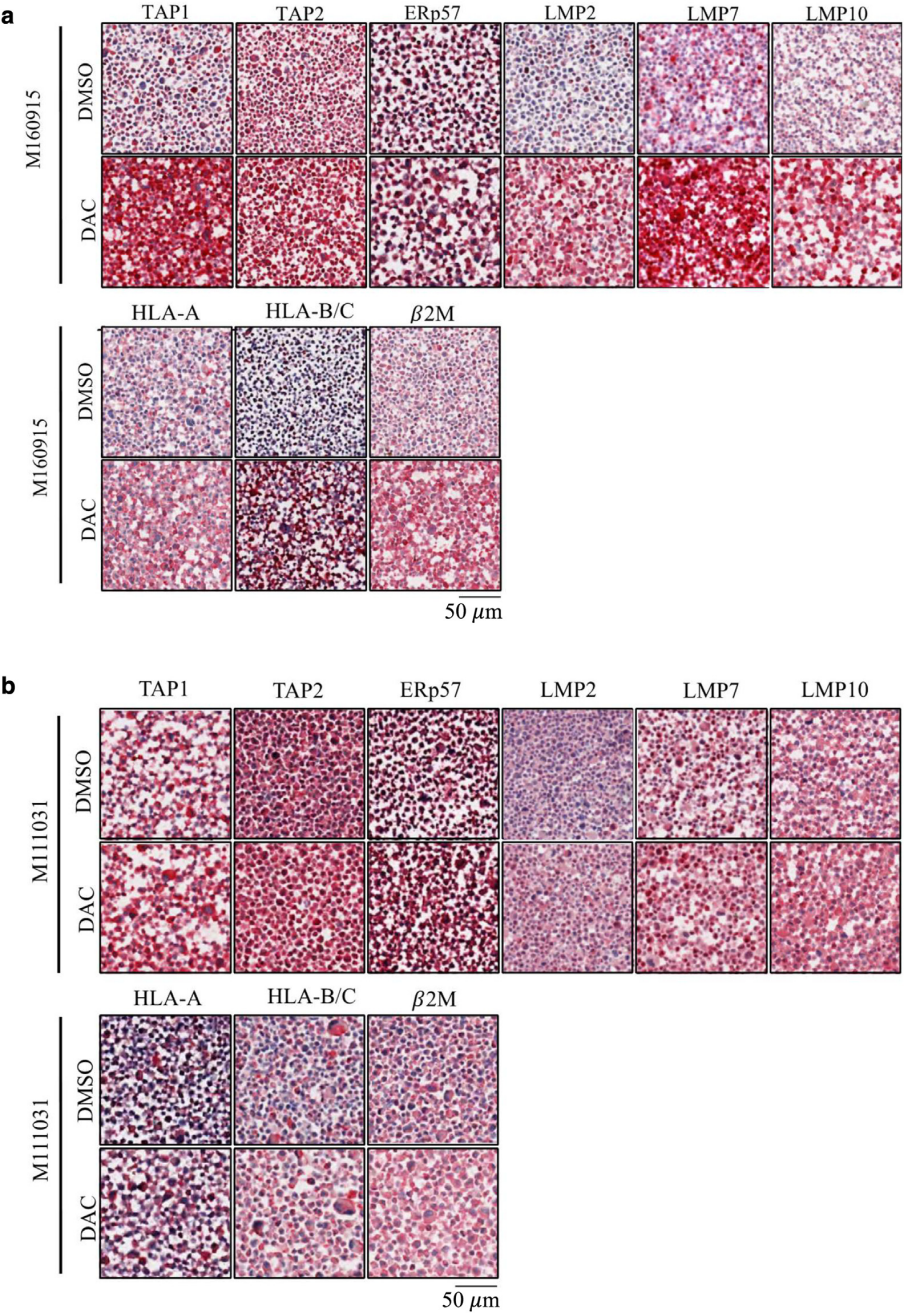
**DNMTi upregulates protein expression of several APM components in patient-derived melanoma cells.** (**a, b**) Representative images of **(a)** M160915 and **(b)** M111031 immunohistochemical samples. Cells were treated with DAC (2.5 μM)± MEK162 (100n M) for 72 hours. Prior staining, formalin-fixed cells were cut (4 μm) and stained with primary antibodies targeting several APM proteins (Supplementary Tables S4 and S5). The scale is indicated below the figure (50 μ m). APM, antigen processing machinery; DAC, decitabine; DNMTi, DNA methyltransferase inhibitor; MEK, MAPK/extracellular signal–regulated kinase kinase.

**Figure 18:**
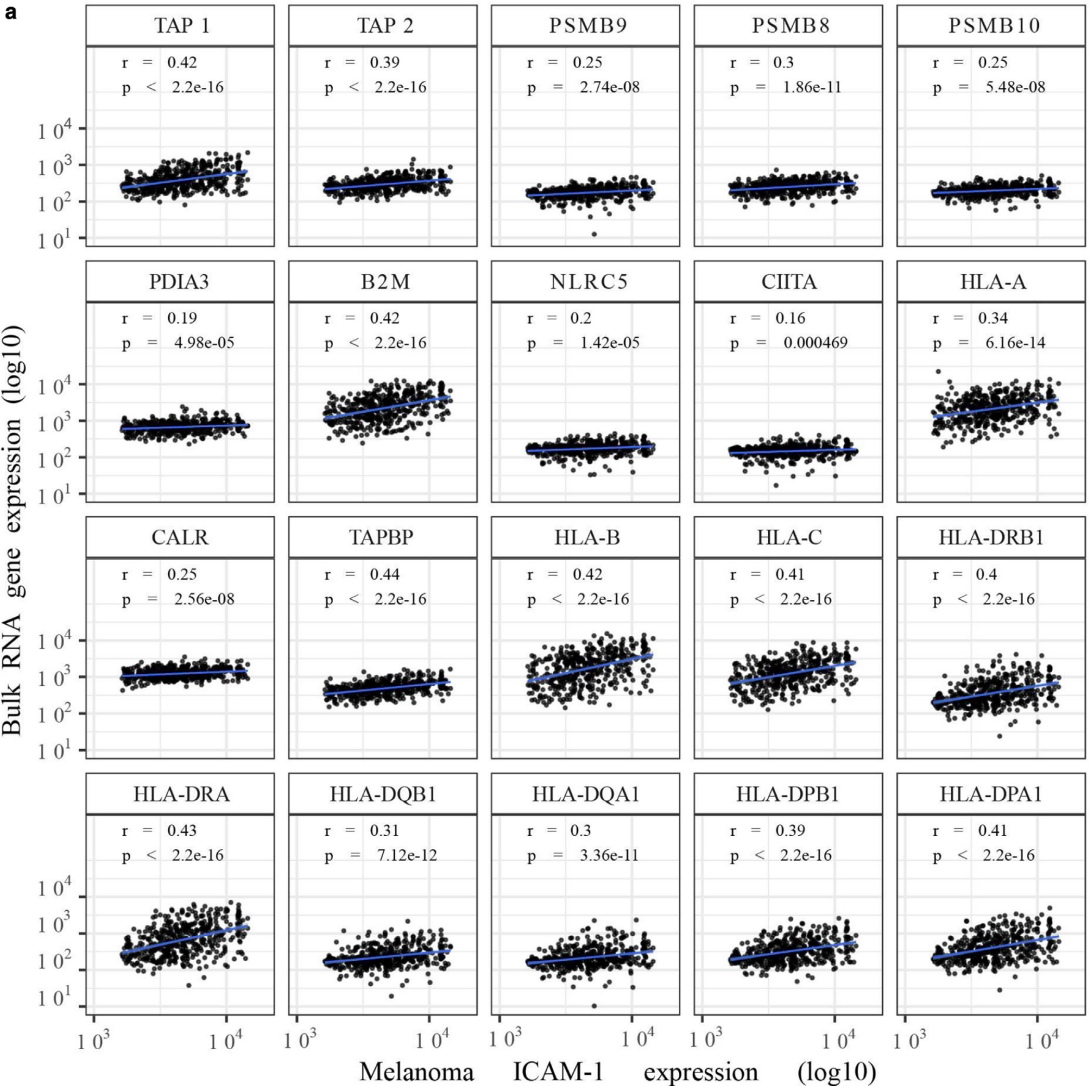
**Melanoma ICAM-1 expression correlates with the extended APM gene signature.** (**a**) Pearson’s correlation coefficient (r) analysis between each gene of the extended APM signature and ICAM-1 expression on melanoma cells in the bulk RNA-sequencing data of the TCGA SKCM. Pearson’s correlation coefficient (r) and correlation *P*-value are depicted (∗*P*≤.05). APM, antigen processing machinery; B2M, β-2-microglobulin; CIITA, class II major histocompatibility complex transactivator; HLA, major histocompatibility complex; PDIA, protein disulfide isomerase; PSMB, proteasome 20S subunit beta; SKCM, skin cutaneous melanoma; TAP, transporter ATP binding cassette subfamily B member; TAPBP, TAP binding protein; TAZ, tazemetostat; TCGA, The Cancer Genome Atlas.

In melanoma, HLA Class I internalization is mechanistically driven by the BRAF^V600E^ mutation (Bradley et al, 2015). Recently, it was shown that DNMTi induces expression of HLA Class I and β-2-microglobulin in melanoma (Di Giacomo et al, 2019). These observations motivated us to test the interactions between DNMTi and MAPKi (in MAPKi-resistant cell lines) or DNMTi only (in MAPKi-sensitive cell lines) in modulating melanoma immunogenicity. To do this, we first examined HLA protein levels in our melanoma cells lines. Consistently, protein levels analysis of DAC- treated cells showed increased expression of HLA class I, HLA-A/B/C and II, HLA-DR/DP/DQ, in most melanoma cell lines (Figure 15a). Importantly, combination of DAC with MEK162 or LGX818 did not further increase their expression, indicating that DNA-methylation is the primary mechanism of tumor immunogenicity downregulation. Next, we further evaluated DNMTi-induced modulation of selected known key components of the antigen-presenting machinery (APM), including chaperones (β-2-microglobulin), immunoproteasome subunits (LMP2/7/10), and antigen-processing specialized proteins (TAP1/2, TAPBP, and ERp57). In line with the HLA profiles, DAC induced upregulation of APM proteins (Figure 15b), and this positively correlated with ICAM-1^high^ expression (Figure 15c), thus indicating a further correlation between ICAM-1^high^ and increased tumor immunogenicity (Figure 15c). We could also show that this upregulation was transcriptionally driven (Figure 16), and further confirmed it by immunohistochemistry analysis (Figure 17). Finally, to determine whether the trend observed in vitro could be identified in vivo, we analyzed once more the SKCM TCGA melanoma dataset, which confirmed positive correlation between ICAM-1^high^ expression on melanoma cells and significant upregulation of other APM components (Figure 18). Taken together, our data support the potential of hypomethylating agents in improving HLA I/II expression and inducing APM upregulation, further expanding our understanding of the epigenetic regulation of immunomodulatory melanoma antigens.

## Discussion

Besides the major genetic drivers leading to melanoma onset and progression, melanoma epigenetic states (and specifically DNA methylation) support the immunoediting-induced selection of low antigenic (and therefore poorly immunogenic) melanoma clones (Dunn and Rao, 2017). In this study, we describe the implications of targeting patient-derived cell lines by simultaneous administration of DNMTi and MAPKi. Our data support the superiority of DNMTi over other epigenetic inhibitors in reshaping a selection of surfaceome markers of advanced melanoma cells, specifically by inducing ICAM-1 upregulation. Our results describing residual DAC-induced ICAM-1 expression in *TP53* loss-of-function cells after p38 inhibition are in line with previous reports that identified a direct modulation of ICAM-1 after administration of demethylating agents (Fazio et al, 2018). This suggests a dual p53- and p38-dependent DNMTi-induced reinforcement of the immune synapse despite MAPKi administration. Besides the surfaceome alterations, we also studied the viability effect of combinatorial DNMTi and MAPKi administration. In this study, we show that DNMTi induced apoptosis also in MAPKi-resistant cells. Mechanistically, disruption of DNA flexibility and alterations of chromatin stability by administration of cytosine analogs are linked to the appearance of DNA-damage foci (Bull et al, 2021; Luijsterburg and van Attikum, 2011). Indeed, prolonged exposure to genotoxic stress eventually triggers an apoptotic response, which improves the presentation of tumor-derived antigens on the surface of professional antigen-presenting cells to innate and adaptive immune cells (Zhou et al, 2019; Zingoni et al, 2017). In this study, we show that in vitro inhibition of melanoma viability was significantly associated with increased DNA-damage foci and expansion of a γH2AX^high^ population, which lead eventually to apoptosis induction in resistant cells. Dual inhibition of BRAF and DNMT has been tested in several patient-based trials but never reached the last phase of clinical testing (Phadke et al, 2015; Zakharia et al, 2017), whereas the triple combination of BRAF inhibitor + MEK inhibitor + DNMTi was never investigated. Taken together, these results support the potential of this triple combination in reducing melanoma proliferation also in double-pathogenic variant cell lines.

Numerous studies tried to delineate the implications of melanoma ICAM-1 expression on its progression. Given its role in mediating homotypic and heterotypic cell interactions, ICAM-1 expression by melanoma cells mediates CD8^+^ T-cell melanoma cell lysis, regulates the formation of melanoma–CD8^+^ T cell–lymph node aggregates, and is superior to the BRAF/NRAS alteration status in predicting response to MAPKi (Hamaï et al, 2008; Krisp et al, 2018; Menares et al, 2019; Straetemans et al, 2015; Yanguas et al, 2018). In contrast, others showed that ICAM-1 upregulation may have detrimental effects in supporting melanoma extravasation across the endothelium and in shaping the metastatic niche (Ghislin et al, 2012; Roland et al, 2007). Nevertheless, to our knowledge, it has not yet been investigated how modulation of melanoma ICAM-1 expression may affect the TME. In this study, we described a positive correlation between melanoma ICAM-1^high^ expression and infiltration of antitumorigenic M1 macrophages and CD4^+^ and CD8^+^ T cells. A recent study described that elevated *ICAM1* mRNA levels correlated with significantly improved survival in patients with stage III/IV melanoma (Hailemichael et al, 2018). Importantly, this correlation was also associated with increased transcription of CXCL9 and CXCL10. Remarkably, DNA-methylation–induced silencing of immune effector genes was shown to impair T-cell trafficking to the tumor site by reducing tumor cell secretion CXCL9 and CXCL10 (Peng et al, 2015; Zou et al, 2020). In patients with melanoma, endogenous secretion of CXCL9 and CXCL10 positively correlates with improved prognosis and overall response by induction of CD8^+^ T-cell recruitment to the tumor site and correlates with improved response to anti–PD-1 and anti–CTLA-4 blockade in vitro (Bedognetti et al, 2013; Chheda et al, 2016; Harlin et al, 2009; Ji et al, 2012; Peng et al, 2012). In this study, we show that coadministration of GUA and MAPKi increased melanoma CXCL9/CXCL10 secretion. Interestingly, the CXCL9/CXCL10 secretion increase was stronger after coadministration of GUA and MAPKi, suggesting a possible addictive interaction between these 2 compounds in regulating melanoma T helper 1 chemokine secretion. However, because downstream effects of CXCL9/CXCL10 signaling are strictly dependent on the splicing variant of the CXCR3 receptor, further experiments should be performed to evaluate DNMTi modulation of this chemokine axis (Reynders et al, 2019).

Besides evaluating the effects of DNMTi on melanoma surfaceome and inflammatory secretome, we described its effect on selected APM components, which previous reports showed to be regulated by direct promoter methylation (Chen et al, 2014; Hasim et al; 2012; Straetemans et al, 2015). Moreover, a recent phase II clinical trial established that administration of GUA to patients with melanoma led to a reduced global DNA methylation, which was associated with reinduction of major histocompatibility complex class I/II and β-2-microglobulin expression (Di Giacomo et al, 2019). In this study, we showed that DNMTi upregulated other APM components, which was even more pronounced in melanoma ICAM-1^high^ cells. Remarkably, previous work highlighted the modulation of HLA I recruitment upon ICAM-1 engagement on target cells. Because the data obtained from the SKCM TCGA were not derived by DNMTi-treated patients, we speculate that melanoma ICAM-1 upregulation may be linked not only to HLA I upregulation but possibly to other key APM components. To our knowledge, melanoma ICAM-1 upregulation positively correlating with an immunogenic melanoma phenotype has not been previously shown; however, this should be further addressed in the future by additional experimental validations (eg, by performing patient-matched melanoma T-cell killing assays)

To date, several clinical trials are currently investigating the potential of combining DNMTi with immune checkpoint blockers or chemotherapeutics for patients with advanced unresectable melanoma (NCT02816021, NCT04250246, NCT02650986, NCT03903458, NCT03765229, NCT05089370, NCT00925132, NCT01876641, NCT04648826, NCT00715793, NCT02608437) (www.clinicaltrialgov.com, last update: May 4, 2024). However, only 1 of these (NCT01876641) is focusing on DNMTi + MAPKi coadministration. In this study, we show that the modulation of the melanoma epigenome by DNMTi acts on 3 key regulators of melanoma immunogenicity (ie, surfaceome, secretome, and APM), thus possibly preventing (or reversing) the chromatin rearrangements that support MAPKi resistance (Haas et al, 2021). Collectively, our results support future efforts in exploring the potential role of DNMTi as adjuvants to MAPK-targeted therapies for the treatment of patients with advanced melanoma.

## Materials and Methods

Supplementary Table S2 provides the detailed reagent list.

### Surfaceome screen of resistant NRAS human melanoma spheroids

For the surfaceome screen, a resistant NRAS Q61R patient-derived melanoma cell line (M160915) was selected on the basis of its resistance to MEK162. Briefly, M160915 cells were seeded (1500 cells per spheroid, 81 spheroids per mold) in home-made spheroid casting agarose micromolds (MicroTissues PetriDish) according to the manufacturer’s instructions. The epigenetic inhibitors DAC (2.5 μM), tazemetostat (3 μM), belinostat (100 nM), and GSK2879552 (250 nM) were administered as single treatment (Vf = 2.5 ml) 4 days after seeding or in combination with MEK inhibitor MEK162 (100 nM) 7 days after seeding. Control spheroids were treated on day 4 with a corresponding dilution of drug solvent (DMSO) and on day 7 with MEK162 (100 nM). On day 10, melanoma spheroids were dissociated with Accumax. Single-cells were counted and stained using the fixable LIFE/DEAD Fixable Near-IR dead-cell staining kit. To analyze the 5 different conditions and reduce technical variation, cells were first stained using anti-CD44 antibodies, coupled with 5 different fluorochromes to generate a treatment-specific barcoding. Cells were pooled and aliquoted into the four 96-well plates of the LEGENDScreen Human phycoerythrin kit (Figure 2 and Supplementary Table S3). Cells were stained according to the manufacturer’s instructions. After staining, cells were fixed, and fluorescence was quantified using the BD LSRFortessa instrument (BD Biosciences). Analysis of 361 surface protein expression was performed by quantification of phycoerythrin fluorescence intensity after classical population gating (forward scatter area vs side scatter area), doublets exclusion (forward scatter area vs forward scatter height), and exclusion of NIR^+^ dead cells. CD44-based debarcoding allowed for assessment of treatment-specific protein modulation (Figure 3 and Supplementary Table S3). For each surface protein, treatment-dependent MFI was expressed as log_2_ fold change of control MFI (MEK162-treated spheroids). Evaluation of mean difference was analyzed by Welch’s *t*-test (significant *P* ≤ .05). Owing to the 1-shot nature of the primary surfaceome screening, no statistical analysis was performed on these data. Following the same experimental approach, surfaceome screen hits were validated in independent subsequent experiments, and statistical significance was tested by unpaired *t*-test.

### RNA extraction, reverse transcription, and quantitative PCR

Total RNA was extracted using the NucleoSpin RNA isolation kit, according to the manufacturer’s instructions. Priming of mRNA was performed using random hexamer primers and reverse transcribed using MultiScribe Reverse Transcriptase. Quantitative PCR was performed according to the manufacturer’s instructions using the KAPA SYBR FAST quantitative Kit on a rotor gene Q system (Qiagen). Samples were always run as duplicates, normalized to GAPDH-relative levels, and expressed as relative fold changes. Supplementary Table S4 provides details on primers’ sequences.

### In silico analysis of TCGA SKCM cohorts

Bulk RNA-sequencing data were obtained from the SKCM repository of TCGA. Data were normalized first by raw read conversion into counts per million using the R/Bioconductor package edgeR (Robinson et al, 2010) and then by further normalization using a set of 20 reference housekeeping genes (Cui et al, 2021). For each sample, the geometric mean expression of these genes was subtracted from the expression of all other genes. Cell types present in the TCGA SKCM data were imputed using CIBERTSORTx (Chen et al, 2018). Counts per million and housing-keeping-gene–normalized bulk RNA-sequencing data were deconvoluted using 2 modes. In the first mode, the cell-type composition of each sample was imputed using the single-cell RNA-sequencing cell type–specific gene expression profiles as previously shown (Tirosh et al, 2016). In the second mode, we performed the deconvolution mentioned earlier while also inferring the cell type–specific expression of ICAM-1 and several gene sets. On the basis of this inferred expression, we divided the TCGA cohort into ICAM-1 high and low expressors by taking the top and bottom quartiles of samples expressing ICAM-1 on malignant cells. Cell-type compositional differences between ICAM-1^high^ and ICAM-1^low^ patient cohorts were assessed by (i) fitting a linear model explaining cell-type fraction as a function of cell type, ICAM-1 category, and their interaction; (ii) computing estimated marginal means for each of the resulting groups; (iii) computing *P*-values for all pairwise comparisons; and (iv) adjusting the *P*-values for multiple comparisons. For the correlation analysis between melanoma ICAM-1^high/low^ and infiltration of immune cells in the TCGA SKCM, we used the LMM2 immune cell gene signature (Prummer et al, 2022). Significant interactions, which indicate a difference within cell type on the basis of melanoma ICAM-1 status, were assessed by computing estimated marginal means for all pairwise comparisons, calculating *P*-values for these comparisons, with *P*-values adjustment using the Benjamini–Hochberg method (significant *P*-value: ∗*P* ≤ .05, ∗∗*P* ≤ .01, and ∗∗∗*P* ≤ .001). For the correlation analysis between ICAM-1 and HLA-I/II expression on malignant cells, expression of HLA-I/II genes was compared with ICAM-1 expression in the bulk TCGA RNA-sequencing data (log10 transformed) using simple linear models. For each gene, a linear model was estimated and Pearson’s correlation coefficient (r) calculated with associated *P*-value. A similar approach was followed for the correlation analysis with IFN-γ signature. Furthermore, a total IFN-γ score was calculated by taking the mean expression of all genes in the signature and correlated with ICAM-1 expression as a continuous variable or in the ICAM-1^high^ versus ICAM-1^low^ TCGA SKCM cohorts. For the comparison between ICAM-1^high^ and ICAM-1^low^ cohorts, a 1-way ANOVA test with posthoc Dunnett’s multiple comparison correction was performed.

### Cell culture and slow-frozen biopsies

Human primary melanoma cell lines and MAPKi-resistant slow-frozen biopsies were obtained from the URPP melanoma biobank (Zurich, Switzerland) upon patient written, informed consent. Human melanoma cell lines were cultured in RPMI 1640 medium supplemented with 10% heat-inactivated foetal calf serum, L-glutamine (2 mM), sodium pyruvate (1 mM), and 1% penicillin/streptomycin in a cell culture incubator (37 °C, 95% oxygen, 5% carbon dioxide).

### Cytokine array

Patient-derived human melanoma cell lines (M160915, M130219) were seeded (750,000 cells/10-cm dish) and treated daily with GUA (Cf = 2.5 μM) and/or MEK162 (100 nM) for 72 hours. Cell culture supernatant was harvested, and secretion of proinflammatory cytokines was detected and quantified using the human inflammatory array membrane kit (ab134003), according to the manufacturer’s instructions.

### HistoGel sample preparation and processing for immunohistochemistry

Patient-derived human melanoma cell lines (M160915, M111031) were seeded (750,000 cells/10-cm dish) and treated daily with DAC (Cf = 2.5 μM) for 72 hours. Cells were harvested, washed, resuspended in a buffered H&E solution (1:24 H&E in PBS + 4% formalin, v/v) and fixed (room temperature, 4 hours). Buffer residues were removed by repeated centrifugation cycles (400 r.p.m, 5 seconds), and cells were resuspended in HistoGel, according to the manufacturer’s instructions. Samples were dehydrated overnight, re-embedded in paraffin blocks, and processed for cutting (4 μ m). Samples were deparaffinized using Bond Dewax Solution, according to the manufacturer’s instructions, and stained using the fully automated advanced staining system BOND RXm and a bond Polymer Refine Red Detection Kit. Supplementary Tables S4 and S5 provides a detailed antibody list. For quantification of protein expression, whole-cell population was divided into 3 classes of protein expression (low expression, medium, or high expression). For each sample, 4 rectangular regions were analyzed. Protein expression was quantified using the free software QuPath2, as previously described (Bankhead et al, 2017). Protein expression evaluation was based on arbitrary units as follows: low, no staining to faint staining; medium, faint-to-medium staining; and high, medium-to-high staining. The low/medium/high expression cut offs were arbitrarily chosen for each marker according to the staining quality and were kept constant across the analysis of the biological and technical replicates. For each sample, constant square regions (3–4 according to the sample size) were randomly selected for the protein expression evaluation. For the final marker expression relative quantification, the mean value of the independent measurements was taken as the final score. Supplementary Tables S4 and S5 provides antibody and protocol details.

### Drug synergy analysis

MAPKi-resistant cell lines were seeded (2000 cells per well, 96-well plate, 90 μl/well) in a 6 × 6 matrix. DAC and/or MAPKi (Cf = 0–10 μM, Vi = 10 μl) were administered 24 and 96 (with fresh medium supplement, 90 μl/well) according to cell line resistance (Figure 6f and Supplementary Table S6). Ten days after seeding, the drug–medium solution was completely replaced with a solution of resazurin reagent and fresh medium (1/10 v/v, Vf = 100 μl). Cells were incubated (37 °C, 1–2 hours), and metabolic activity of viable cells was measured as a function of absorbance according to the manufacturer’s instructions. Drug synergy scores were calculated on the basis of ZIP reference model after outlier detection using the free online software SynergyFinder2.0 (https://synergyfinder.fimm.fi) as previously shown (Ianevski et al, 2019, 2020). Drug synergy scores between −10 and 10 indicate an additive effect.

### Statistical analysis

All statistical tests were performed using GraphPad Prism, version 5 or 8, for Apple (GraphPad Software, www.graphpad.com). Unless differently specified, 3 or more biological replicates for in vitro experiments were generated and shown as mean ± SD. Statistical significance was tested using 1- or 2-way ANOVA or Student’s *t*-test. For each protein analyzed in the initial surfaceome screening, treatment-dependent MFI was expressed as log_2_ fold change of control MFI (MEK162-treated spheroids). Evaluation of mean difference was analyzed by Welch’s *t*-test (significance verified for *P* ≤ .05). For 1-way ANOVA, posthoc Dunnett’s multiple comparison correction was performed. For 2-way ANOVA, posthoc Sidak’s multiple comparison correction was performed. For pairwise comparisons, Tukey’s correction was used. For comparison of 2 groups, unpaired or paired *t*-tests were performed as indicated in the corresponding figure legend. For the surfaceome screen, all treatment combinations were simultaneously processed to avoid any batch effects. For the in silico analysis, gene variance was quantified by Pearson’s correlation coefficient (r), and statistically significant variation in cell population frequency was determined after adjustment of *P*-values for multiple comparisons. For all analysis, *P*-values were defined as follows: ∗*P* ≤ .05, ∗∗*P* ≤ .01, and ∗∗∗*P* ≤ .001.

## Ethics Statement

Human primary melanoma cell lines and MAPK inhibitor–resistant slow-frozen biopsies were obtained from the URPP melanoma biobank (Zurich, Switzerland) upon written, informed consent from patients.

## Data Availability Statement

No large datasets were generated or analyzed during this study. Minimal datasets necessary to interpret and/or replicate data in this paper are available upon request to the corresponding author.

## ORCIDs

Alessandra S. P. Cereghetti: http://orcid.org/0000-0003-1310-5234

Patrick Turko: http://orcid.org/0000-0003-3695-8264

Phil Cheng: http://orcid.org/0000-0003-2940-006X

Stephan Benke: http://orcid.org/0000-0001-5645-6777

Ala’a Al Hrout: http://orcid.org/0000-0001-9130-6774

Andreas Dzung: http://orcid.org/0000-0002-1776-1928

Reinhard Dummer: http://orcid.org/0000-0002-2279-6906

Michael O. Hottiger: http://orcid.org/0000-0002-7323-2270

Richard Chahwan: http://orcid.org/0000-0002-8672-7790

Lorenza P. Ferretti: http://orcid.org/0000-0002-0341-1425

Mitchell P. Levesque: http://orcid.org/0000-0001-5902-9420

## Conflict of Interest

The authors state no conflict of interest.

## Figures and Tables

**Supplementary Table S1:** Gene Signatures for Correlation Analyses

Gene Signature Name	Reference	Gene List
IFN-**γ** expanded gene signature	Ayers et al, 2017	*CD8A*, *GZMA*, *GZMB*, *IFNγ*, *EOMES*, *CXCL9*, *CXCL10*, *TBX21*
Expanded gene set	Ayers et al, 2017	*IL2RG*, *CXCR6*, *CD3D*, *CD2*, *ITGAL*, *TAGAP*, *CIITA*, *HLA*.*DRA*, *PTPRC*, *CXCL9*, *CCL5*, *NKG7*, *GZMA*, *PRF1*, *CCR5*, *CD3E*, *GZMK*, *IFNG*, *HLA.E*, *GZMB*, *PDCD1*, *SLAMF6*, *CXCL13*, *CXCL10*, *IDO1*, *LAG3*, *STAT1*, *CXCL11*
Immunosuppressive TME	Cui et al, 2021	*FAP*, *PDGFRB*, *CD163*, *SIGLEC1*, *IL10*, *CCL2*, *CCL8*, *CCL13*, *INHBA*, *VCAN*, *AXL*, *TWIST2*, *ADAM12*, *COL6A3*, *STC1*, *ISG15*, *BCAT1*, *OLFML2B*
T-effector and IFN-γ signature	Fehrenbacher et al, 2016	*CD8A*, *GZMA*, *GZMB*, *IFNγ*, *EOMES*, *CXCL9*, *CXCL10*, *TBX21*
Six-plex mIF signature	Berry et al, 2021	*PD1*, *PDL1*, *CD8*, *FoxP3*, *CD163*, *Sox10/S100*

Abbreviations: STAT1, signal transducer and activator of transcription 1; TME, tumor microenvironment.

**Supplementary Table S2:** Reagent List

Reagent/Equipment	Catalog Number	Producer
RPMI 1640 medium	R0883-500ML	Sigma
FCS	35-089-CV	Corning
L-glutamine	25030-024	Gibco
Sodium pyruvate	11360-039	Gibco
MicroTissues PetriDish	Z764019	Merck
Decitabine	A10292	Adooq
Guadecitabine sodium	HY-15229	MedChemExpress
Tazemetostat	A12712	Adooq
Belinostat	A21772	Adooq
GSK2879552	A15547	Adooq
MEK162	A11493	Adooq
LGX818	A13226	Adooq
SB203580	A10824	Adooq
Pifithrin-*α*	P4359	Sigma Aldrich
Near-IR dead-cell staining kit	L10119	Invitrogen
LEGENDScreen Human PE kit	700007	BioLegend
Anti-CD44 FITC (clone IM7)	103006	BioLegend
Anti-CD44 BV510 (clone IM7)	103044	BioLegend
Anti-CD44 PacBlue (clone IM7)	103020	BioLegend
Anti-CD44 APC (clone IM7)	103012	BioLegend
Anti-CD44 Alexa Fluor700 (clone IM7)	103206	BioLegend
Anti-*γ*H2A.X Alexa Fluor 647	613407	BioLegend
Annexin V-FITC Apoptosis Detection Kit	ab14085	Abcam
NucleoSpin RNA isolation kit	740955	Macherey-Nagel
MultiScribe Reverse Transcriptase	4311235	Thermo Fisher Scientific
Random hexamer primers	48190011	Thermo Fisher Scientific
KAPA SYBR FAST qPCR Kit	KK4618	KAPA Biosystems
Accumax	A709	Sigma-Aldrich
HistoGel	HG-4000-12	Thermo Fisher Scientific
BOND RXm	49.1501	Leica Biosystems
Bond Polymer Refine Red Detection System	DS9390	Leica Biosystems
Bond Dewax Solution	AR9222	Leica Biosystems
CellTrace CFSE	C34554	Thermo Fisher Scientific
Human Inflammation Antibody Array Membrane	ab134003	Abcam
Resazurin	R7017	Sigma-Aldrich

Abbreviations: CFSE, carboxyfluorescein succinimidyl ester; FCS, fetal calf serum.

The data provided are relative to the Materials and Methods section.

**Supplementary Table S3:** LEGENDScreen Ab Antibody List

Well	Plate 1	Plate 2	Plate 3	Plate 4	Well	Plate 1	Plate 2	Plate 3	Plate 4
A1	Blank	Blank	Blank	Blank	E1	CD15	CD40	CD272	GARP
A2	IgG isotype control	CD244	DLL1	HLA-DR	E2	CD150	CD41	CD198	CD215
A3	CCR10	CD245	DLL4	Ig light chain λ	E3	CD151	CD42b	CCRL2	Lymphotoxin β receptor
A4	CD278	CD25	DR3	IgD	E4	CD154	CD43	CD102	MRGX2
A5	IFN-γ R b chain	CD252	EGFR	IL-28RA	E5	CD156c	CD44	CD104	TMEM8A
A6	IgG1, κ isotype control	CD261	GPR19	integrin β5	E6	CD158e1	CD45	CD124	CD254
A7	CD46	CD262	GPR56	KLRG1	E7	CD16	CD47	CD130	CD318
A8	CD70	CD263	HLA-E	LOX-1	E8	CD161	CD48	CD144	IgG3,k isotype control
A9	CD1a	CD266	HVEM	MICA/MICB	E9	CD162	CD49a	CD152	CD255
A10	CD2	CD268	Ig light chain κ	SUSD2	E10	CD163	CD49b	CD155	SSEA-4
A11	β2M	CD27	IgM	Notch 2	E11	CD164	CD49c	CD158b	IgM, κ isotype control
A12	B7-H4	CD271	CD360	TACSTD2	E12	CD165	CD49d	CD184	Sialyl Lewis X (dimeric)
B1	Cadherin 11	CD275	Integrin α9β1	TIGIT (VSTM3)	F1	CD166	CD5	CD186	TRA-1-81
B2	CD10	CD276	Jagged 2	IgG2b, κ isotype control	F2	CD169	CD50	CD192	CD160
B3	CD100	CD146	Ksp37	C3aR	F3	CD170	CD54	CD197	CD57
B4	CD103	CD279	LAP	CCX-CKR (CCRL1)	F4	CD172a/b	CD55	CD199	CD66b
B5	CD105	CD28	LY6G6D	CD11c	F5	CD172g	CD56	CD209	TRA-1-60-R
B6	CD106	CD29	MERTK	CD129	F6	CD178	CD58	CD217	IgG1, κ isotype control
B7	CD107a	CD290	MSC	CD158	F7	CD179a	CD6	CD230	CD115
B8	CD107b	CD298	MSC,NPC	CD181	F8	CD179b	CD61	CD24	CD201
B9	CD109	CD3	TNAP	CD193	F9	CD18	CD62E	CD243	IgG2a, κ isotype control
B10	CD111	CD30	MUC-13	CD196	F10	CD180	CD62L	CD26	CD120b
B11	CD112	CD300c	NKp80	CD1d	F11	CD182	CD62P	CD269	CD210
B12	CD114	CD309	Notch 1	CD20	F12	CD183	CD63	CD282	CD267
C1	CD116	CD31	Notch3	CD22	G1	CD185	CD64	CD284	CD294
C2	CD117	CD314	Notch 4	CD220	G2	CD19	CD69	CD301	CD49f
C3	CD119	CD317	NPC	CD235ab	G3	CD191	CD73	CD303	CD85
C4	CD11a	CD324	CD352	CD258	G4	CD194	CD74	CD304	CD85d
C5	CD11b	CD325	PSMA	CD274	G5	CD1b	CD79b	CD307e	IgG Fc
C6	CD122	CD328	ROR1	CD319	G6	CD1c	CD8a	CD323	Integrin β7
C7	CD123	CD33	Siglec-10	CD32	G7	CD200	CD80	CD357	XCR1
C8	CD126	CD334	CD328	CD326	G8	CD200R	CD81	CD36	Podoplanin
C9	CD127	CD335	Siglec-8	CD338	G9	CD202b	CD82	CD369	IgG2b, κ isotype control
C10	CD13	CD336	Siglec-9	CD368	G10	CD203c	CD83	CD370	CD132
C11	CD131	CD337	SSEA-5	CD45RA	G11	CD205	CD85	CD371	CD195
C12	CD134	CD34	SUSD2	CD45RB	G12	CD206	CD85k	CD45RO	CX3CR1
D1	CD135	CD340	TCR α/β	CD49e	H1	CD207	CD87	CD51	IgM, κ isotype control
D2	CD137	CD344	TCR γ/δ	CD52	H2	CD21	CD89	CD59	SSEA-3
D3	4-1BBL	CD35	Tim-4	CD66a/c/e	H3	CD213α1	CD8a	CD7	Blank
D4	CD138	CD354	TLT-2	CD85h	H4	CD213α2	CD9	CD71	Blank
D5	CD14	CD360	TM4SF20	CD85	H5	CD218a	CD90	CD84	Blank
D6	CD140a	CD365	TRA-2-49	CD86	H6	CD221	CD93	CD88	Blank
D7	CD140b	CD366	TRA-2-54	CD92	H7	CD223	CD94	CD355	Blank
D8	CD141	CLEC4A	TSLPR	CXCR7	H8	CD226	CD95	erbB3	Blank
D9	CD142	CD36L1	VEGFR-3	Delta opioid receptor	H9	CD227	CD96	FPR3	Blank
D10	CD143	CD38	IgG2a, κ isotype control	Dopamine receptor D1	H10	CD229	CD97	GD2	Blank
D11	CD146	CD39	CD133	EphA2	H11	CD23	CD99	GPR83	Blank
D12	CD148	CD4	APCDD1	FcεRIα	H12	CD231	CXCL16	HLA-A/B/C	Blank

Abbreviation: Ab, antibody.

**Supplementary Table S4:** RT-qPCR Primer List

Target Gene	Forward Primer	Reverse Primer
*ICAM1*	5'-CTTCGTGTCCTGTATGGCCC-3'	5'-ACAGAGGTAGGTGCCCTCAA-3'
*TAP1*	5'-TCAGGGCTTTCGTACAGGAG-3'	5-TCCGGAAACCGTGTGTACTT-3'
*TAP2*	5'-ACTGCATCCTGGATCTCCC-3'	5'-TCGACTCACCCTCCTTTCTC-3'
*LMP2*	5'-TCAAACACTCGGTTCACCAC-3'	5'-GGAGAAGTCCACACCGGG-3'
*LMP7*	5'-CATGGGCCATCTCAATCTG-3'	5'-TCTCCAGAGCTCGCTTTACC-3'
*LMP10*	5'-GTGAAGCCACTAACCCTGGA-3'	5'-TCCAAGCTCTAAGCCTCAGC-3'
*β*2-microglobulin	5'-TCTCTGCTGGATGACGTGAG-3'	5'-TAGCTGTGCTCGCGCTACT-3'
*NLRC5*	5′-TGGGAAGACACTCAGGCTAA-3'	5′-ATCATCGTCCTCACAGAGGTT-3'
*GAPDH*	5'-AACGGATTTGGTCGTATTGGC-3'	5'-TCGCTCCTGGAAGATGGTGATG-3'

**Supplementary Table S5:** IHC Antibody List

Target Protein	Catalog Number	Producer	Dilution	Antigen Retrieval Leica Bond ER2
TAP1	—	Soldano Ferrone, Harvard University	1/500	30', 100 °C
TAP2	—	Soldano Ferrone, Harvard University	1/500	20', 100 °C
LMP2	Ab242061	Soldano Ferrone, Harvard University	1/2000	30', 100 °C
LMP7	Ab180606, clone EPR14482(B)	Abcam	1/1000	30', 95 °C
LMP10	NBP1-30151, clone EPR14902)	Abcam	1/100	30', 100 °C
B2M	—	Soldano Ferrone, Harvard University	1/200	30', 100 °C
HLA-A (HCA2)	—	Soldano Ferrone, Harvard University	1/3000	30', 95 °C
HLA-B/C (HC10)	—	Soldano Ferrone, Harvard University	1/500	20', 100 °C
ERp57	—	Soldano Ferrone, Harvard University	1/100	30', 100°C

Abbreviation: IHC, immunohistochemistry.

**Supplementary Table S6:** Drug Synergy Matrix for DAC and MAPKi Coadministration

Drug Treatment Matrix		DAC											
		1	2	3	4	5	6	7	8	9	10	11	12
MAPKi (MEK162 and/or LGX818)	A												
	B	0/0	1/0	10/0	100/0	1000/0	10,000/0	0/0	1/0	10/0	100/0	1000/0	10,000/0
	C	0/1	1/1	10/1	100/1	1000/1	10,000/1	0/1	1/1	10/1	100/1	1000/1	10,000/1
	D	0/10	1/10	10/10	100/10	1000/10	10,000/10	0/10	1/10	10/10	100/10	1000/10	10,000/10
	E	0/100	1/100	10/100	100/100	1000/100	10,000/100	0/100	1/100	10/100	100/100	1000/100	10,000/100
	F	0/1000	1/1000	10/1000	100/1000	1000/1000	10,000/1000	0/1000	1/1000	10/1000	100/1000	1000/1000	10,000/1000
	G	0/10,000	1/10,000	10/10,000	100/10,000	1000/10,000	10,000/10,000	0/10,000	1/10,000	10/10,000	100/10,000	1000/10,000	10,000/10,000
	H												

Abbreviations: DAC, decitabine; MAPKi, MAPK inhibitor.

0/0: First number is for DAC concentration (0–10,000 nM), and second number is for MAPKi concentration (0–10,000 nM). Pink color denotes replicate 1, and green color denotes replicate 2.

## Footnotes

1 Prummer M, Bertolini A, Bosshard L, Barkmann F, Yates J, Boeva B, et al. scROSHI: robust supervised hierarchical identification of single cells. bioRxiv 2022.
